# Production and properties of adhesin-free gingipain proteinase RgpA

**DOI:** 10.1038/s41598-023-37534-x

**Published:** 2023-07-04

**Authors:** Abu Sayeed M. Mahmud, Christine A. Seers, N. Laila Huq, Lianyi Zhang, Catherine A. Butler, Caroline Moore, Keith J. Cross, Eric C. Reynolds

**Affiliations:** Oral Health Cooperative Research Centre, Melbourne Dental School, Bio21 Institute, The University of Melbourne, Parkville, VIC 3010 Australia

**Keywords:** Enzymes, Proteases

## Abstract

The Arg-specific gingipains of *Porphyromonas gingivalis* RgpA and RgpB have 97% identical sequences in their catalytic domains yet their propeptides are only 76% identical. RgpA isolates as a proteinase–adhesin complex (HRgpA) which hinders direct kinetic comparison of RgpA_cat_ as a monomer with monomeric RgpB. We tested modifications of *rgpA* identifying a variant that enabled us to isolate histidine-tagged monomeric RgpA (rRgpAH). Kinetic comparisons between rRgpAH and RgpB used benzoyl-l-Arg-4-nitroanilide with and without cysteine and glycylglycine acceptor molecules. With no glycylglycine, values of *K*_m_, *V*_max_, *k*_cat_ and *k*_cat_/*K*_m_ for each enzyme were similar, but with glycylglycine *K*_m_ decreased, *V*_max_ increased and *k*_cat_ increased ~ twofold for RgpB but ~ sixfold for rRgpAH. The *k*_cat_/*K*_m_ for rRgpAH was unchanged whereas that of RgpB more than halved. Recombinant RgpA propeptide inhibited rRgpAH and RgpB with *K*_i_ 13 nM and 15 nM *K*_i_ respectively slightly more effectively than RgpB propeptide which inhibited rRgpAH and RgpB with *K*_i_ 22 nM and 29 nM respectively (*p* < 0.0001); a result that may be attributable to the divergent propeptide sequences. Overall, the data for rRgpAH reflected observations previously made by others using HRgpA, indicating rRgpAH fidelity and confirming the first production and isolation of functional affinity tagged RgpA.

## Introduction

**Figure 1:**
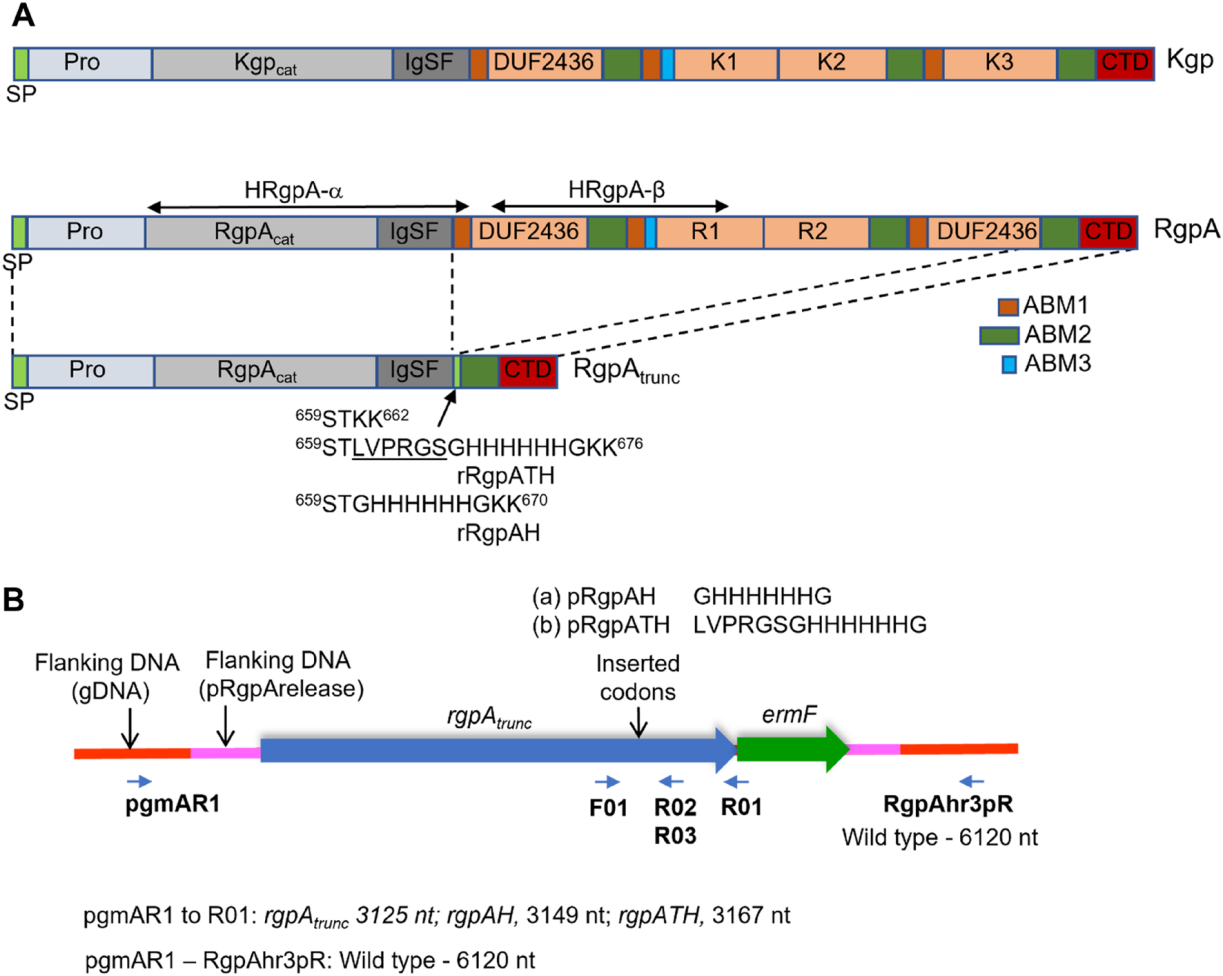
Design for production of recombinant RgpA proteins. (**A**) Schematic of Kgp and RgpA precursor polyproteins and sequences incorporated in the rRgpA truncated precursor (approximately to scale). SP, signal peptide; Pro, propeptide; Kgp_cat_ and RgpA_cat_ catalytic caspase-fold-like domains; IgSF, Ig superfamily fold domain; ABM1 (brown), ABM2 (green), ABM3 (blue), adhesin binding motifs 1, 2 and 3; DUF2436, a domain of unknown function; K1, K2, K3, R1, R2, cleaved adhesin domains; CTD, carboxy-terminal T9SS signal domain; Site of insertion of STKK and His-tagged sequences are indicated. (**B**) Schematic of recombinant *rgpA* loci indicating nucleotides in the recombination cassettes (pRgpArelease, pRgpAH, pRgpATH) and genome flanking the recombination point (gDNA). The approximate site of oligonucleotide annealing for SOE-PCR (F01, R02, R03) are indicated. The oligonucleotides used for genome recombination confirmatory PCR (pgmAR1, R01, RgpAhr3pR) and amplicon sizes are also indicated.

*Porphyromonas gingivalis* is a pathogenic bacterium that has a significant role in the progression of chronic periodontal disease. The cell-surface proteases RgpA, RgpB, and Kgp, collectively known as the gingipains, account for 85% of the general proteolytic activity exhibited by this organism^1^ and are implicated as major virulence factors in many studies^2–4^. The *rgpA* and *kgp* genes respectively encode Arg-specific RgpA (1706 residue) and the Lys-specific Kgp (1732 residue) proteinase–adhesin polyproteins and require proteolytic processing at multiple sites to generate mature proteinase–adhesin complexes^5–7^. The RgpA and Kgp precursors are each comprised of (from N-terminus to C-terminus) a leader peptide, a propeptide domain, a catalytic domain, a hemagglutinin/adhesin domain, and the C-terminal outer membrane secretion signal (CTD) domain^6,8–10^ (Fig. 1). RgpA and Kgp proteinase–adhesin sub-fragments remain non-covalently associated on the cell surface of most *P. gingivalis* strains examined to date^5,11^. Combined, this non-covalent and membrane association has hindered the purification of RgpA_cat_ and Kgp_cat_ proteinases free of adhesin domains. C-terminal to the RgpA_cat_ and Kgp_cat_ domains is the ABM1 domain (adhesin-binding motif 1)^8,12^. Modification of the ABM1 domain of Kgp results in the release of Kgp_cat_ into the *P. gingivalis* culture medium from where it can be isolated^13,14^. Whether modification to the RgpA ABM1 domain would similarly result in the release of RgpA_cat_ from the *P. gingivalis* cell surface has not been tested. There are three ABM1 motifs in each of the RgpA and Kgp precursors, as well as three additional adhesin binding motifs dubbed ABM2, which may be the intra-complex ABM1 binding partner^12^.

RgpB is not adhesin-associated and is a monomer anchored to the outer membrane from which it is difficult to purify. However, *P. gingivalis* strain HG66 is unusual in that it releases RgpB into the culture fluid from where it can be readily isolated^15^. The determined structure of RgpB of strain HG66 revealed a caspase-like fold containing the active site followed by an immunoglobulin-like domain^16^. The RgpA and RgpB proteinase catalytic domains share 97% identity across the caspase-like folds, whereas the N-terminal propeptides and immunoglobulin-like domains share only 76% and 52% identity, respectively^8,17^. RgpA is isolated as HRgpA (high molecular weight RgpA) from the culture fluid of stationary phase cultures. HRgpA consists of the co-purified Tyr^228^–Arg^688^ α-catalytic domain and the adhesin Ser^720^–Arg^1081^ β-domain^10,11^. Biochemical characterization of HRgpA and RgpB has indicated a similar propensity to cleave amide substrates but differences in propensity to cleave protein substrates with changes in pH^15^. RgpB is more tolerant of pH, showing maximum activity with azocasein substrate at pH 9.0, whereas HRgpA had maximal activity at pH 7.5^15^. Peptide substrate specificities also differ between HRgpA and RgpB which translates to diverse *k*_*cat*_ and *K*_*m*_^18^. Some monomeric *P. gingivalis* strain HG66 RgpA_cat_ has been isolated from stationary phase *P. gingivalis* HG66 culture supernatant during isolation of RgpB^18^. This was possible because, unlike RgpB, RgpA_cat_ did not bind to a DE-52 cellulose column resin under the same conditions, which further highlights that there is diversity in the physiochemical properties of RgpA_cat_ and RgpB. HRgpA and RgpA_cat_ display similar *K*_*m*_ values against selected substrates with HRgpA more efficient at fibrinogen degradation, an observation suggested to be due to the adhesin domain binding fibrinogen and increasing proteinase access to the substrate^18^.

Inhibitors of the gingipains are being investigated for use clinically. The inhibitory potential of recombinant RgpA and RgpB propeptides against HRgpA and RgpB has been demonstrated^13,19,20^. However, the inhibitory potential of the recombinant propeptides against purified RgpA_cat_ monomer is yet to be determined. In this study, we have developed a method for the secretion of RgpA_cat_ into the culture fluid of *P. gingivalis* strain W50, from where it can be readily purified and used for activity and inhibition studies.

## Results

### Design of a recombination cassette for the production of extracellular RgpA_cat_ proteinase

To obtain purified, functional RgpA_cat_ free of adhesin residues, a strategy was devised to modify the *P. gingivalis* strain W50 *rgpA* gene, deleting the majority of the adhesin-coding DNA whilst retaining codons for the CTD secretion signal. The suicide plasmid pRgpArelease contained the modified *rgpA* gene (*rgpA*_*trunc*_) encoding a 790 residue recombinant RgpA. The rRgpA (from N-terminus to C-terminus) has a leader peptide (^1^M-A^23^), prodomain (^24^Q-R^227^), catalytic domain (^228^Y-P^578^), and immunoglobulin fold domain (^579^T-N^658^), followed by a serine, threonine, and two lysine residues (^659^STKK^662^), then, ABM2 and CTD domains (^663^A–^721^A and ^722^D–K^790^) which are equivalent to residues ^1579^A–^1706^ K of the wild-type RgpA (Fig. 1). The two lysine residues ^661^KK^662^ were included as targets for Kgp cleavage for protein release from the cell surface. The C-terminus of ABM2 is the anchor point attaching the recombinant protein to the *P. gingivalis* outer membrane and provides a spatial buffer between the Kgp target ^661^KK^662^ and the cell surface. Downstream of *rgpA*_*trunc*_ in pRgpArelease is *ermF* to serve as a recombination marker and flanking *rgpA*_*trunc*_ and *ermF* are sequences homologous to sequences flanking *rgpA* in the *P. gingivalis* W50 genome (Fig. 1).

The pRgpArelease was electroporated into an *rgpA* deletion strain already available in the laboratory, but despite numerous attempts, transformants were not obtained. However, when the plasmid was electroporated into the *P. gingivalis* W50 wild-type strain, erythromycin-resistant colonies were obtained. The correct recombinants with *rgpA*_*trunc*_ were identified by PCR using the combination of a flanking primer that annealed upstream of the *rgpA* recombination point (2121839–2121864 in the W83 genome^21^ (pgmAR1) and a primer that annealed within *ermF* (R01) (Fig. 1). Oligonucleotide sequences and PCR amplicons indicating recombination events are shown in supporting information in S1 Table and Fig. S1, respectively. A selected clone was designated ECR803. The ECR803 and the various *P. gingivalis* mutants used in this study are described in Table 1.

**Table 1:** *P. gingivalis* strains produced and used in this study.

Clone name	Background strain	Recombinant RgpA	Genotype	Antibiotic resistance^a^	Reference
ECR368	ECR364	–	*kgp*_DABM1_	Em	^13^
W501	W50	–	*rgpA::erm*	Em	^35^
W50B	W50	–	*rgpB:: tetQ*	Tc	^10^
KDP136	ATCC 33277	–	*rgpA::erm, rgpB::tetQ, kgp::cat*	Tc, Em, Cm	^36^
ECR803	W50	rRgpA	*rgpA*_trunc_, *ermF*	Em	This study
ECR804	ECR803	rRgpA	*rgpA*_*trunc*_*, ermF rgpB::tetQ*	Em, Tc	This study
ECR805	W50B	rRgpA	*rgpA*_*trunc*_*, ermF rgpB::tetQ*	Em, Tc	This study
ECR806	W50BK	rRgpA	*rgpA*_*trunc*_*, ermF, rgpB::tetQ, kgp::cepA*	Tc, Ap	This study
ECR821	W50B	rRgpATH	*rgpB::tetQ, rgpATH, ermF*	Tc, Em	This study
ECR822	W50BK	rRgpATH	*rgpATH, ermF, rgpB::tetQ, kgp::cepA*	Tc, Em, Ap	This study
ECR832	W50B	rRgpAH	*rgpAH, ermF rgpB::tetQ*	Tc, Em	This study
ECR833	ATCC 33277	–	*rgpA::cat*	Cm	This study
ECR834	ATCC 33277	–	*rgpB::tetQ*	Tc	This study
ECR835	ECR833	–	*rgpB::tetQ, rgpA::cat*	Tc, Cm	This study

^a^Em, erythromycin; Tc, tetracycline; Cm, chloramphenicol; Ap, ampicillin.

### Soluble, functional adhesin-free rRgpA_cat_ could be identified in the ***P. gingivalis*** culture supernatant

**Figure 2:**
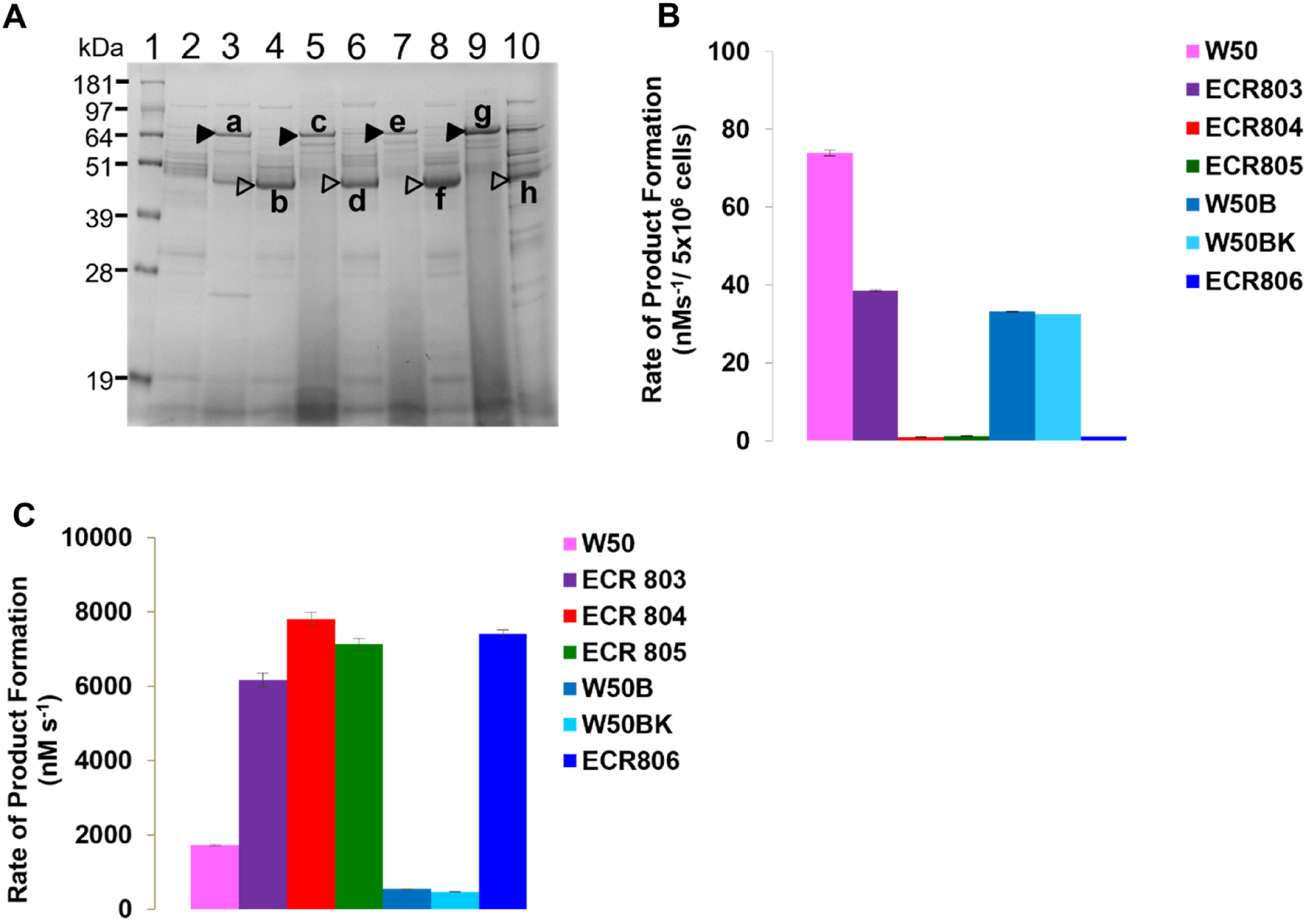
Protein production in pRgpArelease-transformed *P. gingivalis*. (**A**) SDS-PAGE of the VFSN proteins of *P. gingivalis* pRgpArelease transformed mutants harvested at day 1 (D1) and day 3 (D3) of incubation. Lanes 2, 4, 6, 8, 10 D3 VFSN; lanes 3, 5, 7, 9, D1 VFSN. Lane 1, strain W50; Lanes 3–4, ECR803; lanes 5–6, ECR804; lanes 7–8, ECR805; lanes 9–10, ECR806. The closed arrowheads indicate the pro-rRgpA, and the open arrowheads indicate the processed rRgpA, as indicated by LC–MS analysis of these bands a–h (S2–S9 Tables). The sizes of the MW standards are shown on the left. (**B**) The D3 whole-cell and (**C**) D3 VFSN Arg-gingipain activity of *P. gingivalis* pRgpArelease transformed mutants and parent strains. ECR803, W50 parent strain; ECR804, RgpB-null, ECR803 parent; ECR805, RgpB-null, W50B parent; ECR806, W50BK parent, RgpB-null and Kgp-null. The original gel for panel (a) is presented in Supplementary Fig. 7.

*P. gingivalis* ECR803 was inoculated into BHI broth and grown for 3 days with aliquots harvested after one day (D1) and 3 days (D3) of incubation. Cells and extracellular vesicles were removed by centrifugation and the vesicle-free supernatant (VFSN) was examined by SDS-PAGE. ECR803 VFSN at D1 contained a band with protein(s) that migrated slightly slower than the 64 kDa size standard (band “a” Fig. 2) that was not present on D3. Within the D3 sample a band of ~ 45 kDa was evident that was not as prominent in the D1 sample (band “b” Fig. 2). LC–MS analysis of trypsin-digested excised protein band “a” revealed peptides from the RgpA propeptide as well as the catalytic domain, with the most N-terminal, identified peptide being ^66^G-K^80^ and the most C-terminal ^649^E-K^661^ (S2 Table). RgpA ^64^G-K^661^ has a determined mass of 66,135 Da, correlating with the SDS-PAGE band “a” size. Thus, the recombinant *P. gingivalis* is releasing an adhesin-free RgpA prodomain-bearing precursor (pro-rRgpA) into the culture fluid. Band “b” LC–MS analysis gave ^228^Y-K^234^ as the most N-terminal peptide identified and ^649^E-K^661^ as the most C-terminal identified peptide (S3 Table). RgpA ^228^Y-K^661^ has a calculated mass of 47,656 Da, correlating with the SDS-PAGE band “b” size, indicating pro-rRgpA had been processed to give rRgpA_cat_. Arg-protease activity assays conducted using *N*-benzoyl-l-Arg-4-nitroanilide (BApNA) as substrate showed that at D3 ECR803 had 50% of the whole cell activity of the parent strain W50 but greater than threefold more Arg-gingipain activity in the VFSN (Fig. 2), confirming ECR803 produces soluble, active rRgpA_cat_.

### RgpB and Kgp are not essential to produce active rRgpA_cat_

To determine if RgpB influences rRgpA_cat_ release and activation, the *rgpB* gene of ECR803 was inactivated using a *tetQ* gene cassette to produce *P. gingivalis* ECR804 (Table 1). A counter-mutant was also made in strain W50B which already had a *tetQ* point insertion in *rgpB*^10^. W50B was transformed with pRgpArelease, producing strain ECR805. S1 Fig shows PCR confirmation of genome recombination. SDS-PAGE analysis of strain ECR804 and ECR805 VFSN revealed a ~ 67 kDa band in the D1 VFSN of each (bands “c” and “e” respectively Fig. 2) with peptides spanning ^56^F-K^661^ identified using LC–MS (S4 Table and S5 Table). At D3 ECR804 and ECR805 VFSN had bands of ~ 47 kDa (bands “d” and “f” respectively, Fig. 2), which LC–MS indicated were mature rRgpA_cat_, with peptides identified spanning ^228^Y-K^661^ (S6 Table and S7 Table, respectively). Arg-specific protease assay showed negligible whole-cell activity for ECR804 and ECR805 whilst ECR803 had whole-cell activity equivalent to that of W50B. This indicated that the whole-cell activity of ECR803 was likely due to RgpB. In the D3 VFSN, ECR804 and ECR805 exhibited 14-fold more activity than in the W50B parent strain (Fig. 2). Thus, recombinant RgpA as pro-rRgpA_cat_ was released from ECR804 and ECR805, and the prodomain was removed in an RgpB-independent manner.

To determine if pro-rRgpA_cat_ release from the cell and maturation was dependent on Kgp activity, pRgpArelease was transformed into *P. gingivalis* W50BK, an *rgpB::tetQ*, *kgp::cepA* (RgpB-null, Kgp-null) mutant, producing strain ECR806. No whole cell Arg-specific activity was detectable for ECR806, but on D3 of incubation, VFSN Arg-specific proteinase activity was 16-fold more than that of the W50BK parent. LC–MS analysis revealed that ECR806 VFSN contained pro-rRgpA_cat_ in the D1 VFSN (band “g” Fig. 2, S8 Table) and processed rRgpA_cat_ at D3 (band “h” Fig. 2, S9 Table), as per the other strains. However, LC–MS analysis of band “g” revealed peptides that spanned ^56^F-K^710^ indicating rRgpA was cleaved within the ABM2 sequence, C-terminal to the ^661^KK^662^ target site. The most C-terminal peptide identified was ^703^YTAGVSPK^710^ (S2 Fig), but being a tryptic peptide, ^710^K cannot be definitively assigned as the C-terminus of the protein. However, it does indicate that rRgpA_cat_ is released from ECR806 by cleavage within the sequence ^710^KVCVDYIPDGVA^721^ by a non-Kgp proteinase.

### A thrombin-cleavage site in histidine-affinity tagged rRgpA_cat_ is vulnerable to cleavage

Having demonstrated successful rRgpA_cat_ production the pRgpArelease plasmid was then modified so that rRgpA would be produced with a His-tag to facilitate purification. Upstream of the ^661^KK^662^ codons we inserted the codons for a thrombin cleavage site followed by Gly-His-tag-Gly residues (LPVRGSGHHHHHHG, pRgpATH). The thrombin cleavage site was included to facilitate later removal of the His-tag from the proteinase. A plasmid without the thrombin cleavage site codons insertion but with Gly-His-tag-Gly codons (GHHHHHHG, pRgpAH) was also produced (Fig. 1). Plasmid pRgpATH was transformed into *P. gingivalis* strain W50B and into *P. gingivalis* W50BK, whilst pRgpAH was transformed into *P. gingivalis* W50B. The W50B and W50BK *P. gingivalis* transformed with pRgpATH were designated ECR821 and ECR822, respectively. The *P. gingivalis* W50B pRgpAH transformant was designated ECR832 (Table 1).

**Figure 3:**
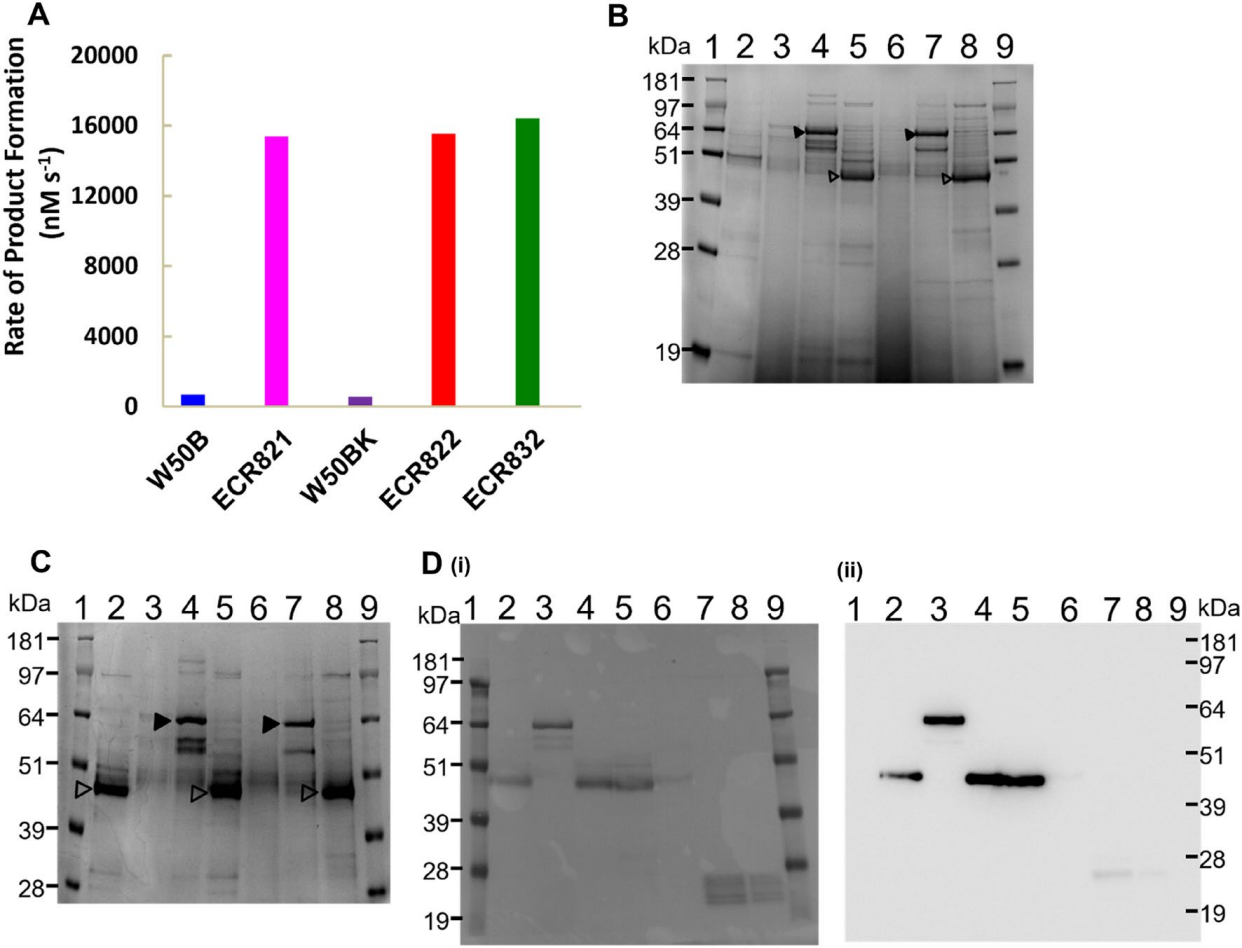
Proteins in pRgpATH and pRgpAH transformed *P. gingivalis* VFSN. (**A**) Arg-specific proteolytic activity in *P. gingivali*s D3 VFSN. (**B**) SDS-PAGE of VFSN proteins. Lane 2, W50B D3; lanes 3–5, ECR821 D1, D2, D3 respectively; lanes 6–8, ECR822 D1, D2, D3 respectively. (**C**) SDS-PAGE of VFSN proteins. Lane 2, ECR803 D3 VFSN; lanes 3–5, ECR821 D1, D2, D3 respectively; lanes 6–8, ECR832 D1, D2, D3, respectively. Bands indicated by closed arrowheads or open arrowheads indicate pro-rRgpAs and mature rRgpAs respectively. (**D**) Western blot of proteins in the VFSN of pRgpAH transformed *P. gingivalis* strain W50B. (i) Ponceau S-stained membrane and (ii) anti-His-tag western blot*.* Lanes 1 and 9, molecular mass standards; Lane 2, colony 1 D3 VFSN; lanes 3–5, colony 2 (designated ECR832) sampled at D1, D2, and D3 respectively; lane 6, colony 3 D3 VFSN; lane 7–8, His-tagged positive control protein, recombinant RgpA propeptide (rRgpA-PP, unpublished). The rRgpA-PP was produced and purified using the same system as rRgpB-PP^13^ with substitution of the *rgpA* codons for the RgpA propeptide in the expression vector. The MW of standards are indicated on the left. The original gels and blot for panels (**B**, **C**, and **D**) are presented in Supplementary Fig. 7.

The Arg-gingipain activities in the VFSN and on the whole cells of both ECR821 and ECR822 mutants were measured after 3 days of incubation. No substantial Arg-gingipain activities were found on the whole cells but approximately 30-fold more Arg-gingipain activities were present in the ECR821 and ECR822 VFSN than in VFSN of the respective parent strain and the SDS-PAGE profiles were also similar (Fig. 3). This indicated the successful production and release of rRgpATH by these strains and that Kgp has no essential role in the production and release of rRgpATH from the surface of *P. gingivalis*. However, attempts to isolate rRgpATH from ECR821 and ECR822 VFSN using Ni-affinity chromatography failed. LC–MS analysis of trypsin-digested rRgpATH VFSN protein bands isolated from SDS-PAGE confirmed the correct rRgpATH N-termini but failed to identify any peptides with a His-tag sequence. The identified C-terminal peptides (^654^TINTNSTLVPR^664^) ended at the Arg residue within the thrombin cleavage site (LVPRGS) (S3 Fig). It is likely that due to the cleavage at the Arg-residue of the thrombin cleavage site, the His-tags were not present in the rRgpATH proteins, thus explaining the failure of the nickel-affinity purification protocol.

### Histidine-tagged rRgpA (rRgpAH) can be purified from ECR832 culture supernatant

ECR832 VFSN Arg-gingipain activity was 30-fold higher than that of the W50B parent strain, indicating the production and release of rRgpAH (Fig. 3). VFSN from D1, D2, and D3 ECR832 cultures were examined using denaturing SDS-PAGE and revealed proteins consistent with pro-rRgpAH that matured over time to produce rRgpAH. The presence of the His-tag in rRgpAH was confirmed by western blot analysis using anti-His-tag antibodies (Fig. 3) and LC–MS analysis (S4 Fig).

Pilot analyses indicated rRgpAH could be captured on both nickel-affinity and cobalt-affinity (TALON) resin but that TALON resin gave better binding. Therefore, TALON resin was used to purify rRgpAH. Filtered ECR832 culture supernatant was mixed with TALON resin and packed into an open column allowing unbound proteins to flow through. The resin was washed, then the bound proteins were eluted using 150 mM imidazole. Eluates were concentrated using centrifugation filter units with a 5 kDa cut-off before further purifying using gel filtration. No Lys-specific proteinase activity was detected in the final product indicating no Kgp contamination (data not shown). Based on the total units of proteolytic activity in the culture fluid and the final gel filtration fractions, 60.5% of rRgpAH was recovered. The final yield of rRgpAH was 5.4 mg from 1 L of starting culture.

The rRgpAH was assessed by mass spectrometry using a Waters Acquity H-class UPLC coupled to a Vion IMS QTOF mass spectrometer (Milford, MA, USA). The expected mass of rRgpAH with both C-terminal lysine (^669^KK^670^) was 48,721.97 Da, or with one C-terminal lysine 48,593.79 Da. However, the deconvoluted mass of the main rRgpAH peak was 48,463.0 Da (S5 Fig), corresponding to the theoretical mass of rRgpAH with both C-terminal lysine residues removed (48,465.62 Da). The deconvoluted spectrum had a small shoulder indicating some proteins were present of higher mass consistent with rRgpAH with one or two C-terminal Lys residues. LC–MS analysis of trypsin-digested rRgpAH enabled the identification of a C-terminal peptide ^654^TINTNSTGHHHHHHGK^669^, indicating that at least some of the rRgpAH retained C-terminal Lys residues (S10 Table).

### The effect of cysteine and glycylglycine on gingipain kinetics

Cysteine and glycylglycine enhance gingipain activities^15,22^. Glycylglycine participates in transpeptidation enhancing *p*-nitroaniline product release from small molecule chromogenic substrates such as BApNA and Gly-Pro-Lys-*p*-nitroanilide (GPKNA)^23^. Cysteine is considered to function in the reduction of oxidized gingipain active site Cys residues but could also be an acceptor in transpeptidation. The activities of purified rRgpAH as well as RgpB and rKgp (purified as described previously^13^) were compared in the presence of varied amounts of cysteine and glycylglycine. Using 20 mM cysteine in the assay, rRgpAH, RgpB, and rKgp activities each increased by approximately five-fold relative to no cysteine addition, a response which increased a further two-fold when the cysteine concentration was increased to 200 mM. However, the enzyme responses to glycylglycine differed, with RgpB only half as responsive to glycylglycine addition as rRgpAH or rKgp. Highest enzyme activities were realized in the presence of both cysteine and glycylglycine (Table 2).

**Table 2:** rRgpAH, RgpB, and rKgp activity fold changes with cysteine addition in the presence or absence of glycylglycine.

Additives	Concentrations used (mM)	Fold activity change with additives		
		rRgpAH	RgpB	rKgp
Cysteine Glycylglycine	0 or 20 0	5.2	5.1	5.6
Cysteine Glycylglycine	0 or 200 0	10	9	11.8
Cysteine Glycylglycine	20 or 200 0	1.9	1.8	2.2
Cysteine Glycylglycine	0 300 (Arg), 180 (Lys)	10.8	5.7	12.8
Cysteine Glycylglycine	0 or 20 300 (Arg), 180 (Lys)	3.2	3.1	2.3
Cysteine Glycylglycine	0 or 20 0 or 300 (Arg), 0 or 180 (Lys)	34	17.6	29.5
Cysteine Glycylglycine	20 or 200 300 (Arg), 180 (Lys)	30	16.2	28.5

The kinetics of the enzymes in varied additive environments were calculated using the assumption that the purified enzyme samples contained only active protein molecules. Although calculation of parameters such as *k*_*ca*t_ may thus be an underestimate, comparison of relative activities of the enzymes in the different environments can still be made. The *k*_*cat*_ of rRgpAH and RgpB were similar without glycylglycine in the assay, but *k*_cat_ increased ~ sixfold for rRgpAH and ~ twofold for RgpB in the presence of glycylglycine. This suggests rRgpAH is better able to use glycylglycine as an acceptor than RgpB. However, the rRgpAH and RgpB *K*_m_ for BApNA also changed when glycylglycine was added to the assay (Table 3). The net effect was that the catalytic efficiency (*k*_cat_/*K*_m_) of rRgpAH was the same with or without glycylglycine but RgpB* k*_cat_/*K*_m_ reduced 2.7-fold upon glycylglycine addition (Table 3). The *K*_m_ of purified rKgp for GPKNA substrate was 39 ± 2.4 µM with *k*_cat_ 5.4 ± 0.04 s^−1^ in the absence of glycylglycine whereas in the presence of 180 mM glycylglycine; the *K*_m_ was determined to be 202 ± 18 µM with* k*_cat_ 22.6 ± 0.7 s^−1^. Overall, as with rRgpAH, the catalytic efficiency of rKgp as indicated by *k*_cat_/*K*_m_ was unchanged upon glycylglycine addition.

**Table 3:** The *k*_cat_ and *k*_cat_/*K*_m_ ratio of purified gingipains in the presence and absence of glycylglycine (20 mM cysteine in each assay).

	Property^a^	rRgpAH	RgpB	rKgp
Plus glycylglycine^b^ with 5 nM enzyme	K_m_ (µM)	72 ± 3.5	52 ± 2.3	202 ± 18
	*V*_max_ (nM s^−1^)	202 ± 2	70 ± 0.5	113 ± 3.5
	*k*_cat_ s^−1^	40.4 ± 0.3	14 ± 0.1	22.6 ± 0.7
	*k*_cat_/K_m_ (s^−1^ M^−1^)	5.6 × 10^5^	2.7 × 10^5^	1.1 × 10^5^
No glycylglycine With 1 nM enzyme	K_m_ (µM)	13.5 ± 1.5	10.2 ± 1.1	39 ± 2.4
	*V*_max_ (nM s^−1^)	7.0 ± 0.1	7.4 ± 0.1	5.4 ± 0.04
	*k*_cat_ (s^−1^)	7.0 ± 0.7	7.4 ± 0.1	5.4 ± 0.04
	*k*_cat_/K_m_ (s^−1^ M^−1^)	5.1 × 10^5^	7.2 × 10^5^	1.4 × 10^5^

^a^In the absence of active site titration confirming enzyme concentration *k*_*cat*_ is presumptive and assumes all enzyme molecules applied to the assay are active. Enzyme concentrations are based upon absorbance at 280 nm wavelength and extinctions coefficients assuming all cysteine are reduced. Extinction coefficient (M^−1^ cm^−1^): rRgpAH 48360, RgpB 49850, Kgp 105090.

^b^Glycylglycine was 300 mM in the rRgpAH and RgpB assays and 180 mM in the rKgp assay.

### The propeptides of RgpA and RgpB are inhibitory against rRgpAH

The inhibitory potential of recombinant RgpA and RgpB propeptides (rRgpA-PP, rRgpB-PP)^13^ against purified rRgpAH and RgpB activities was determined. The *K*_i_ for rRgpA-PP against rRgpAH was 13 nM with a 95% confidence interval (CI) of 12–14 nM and the *K*_i_ against RgpB was 15 nM with a 95% CI of 14–16 nM (*p* > 0.05). However, the *K*_i_ of rRgpB-PP against each enzyme was higher (*p* < 0.0001) at 22 nM (95% CI of 18 to 26 nM) against rRgpAH and 29 nM (95% CI of 26 to 32 nM) against RgpB. Thus, the rRgpA-PP was more efficient at inhibiting both rRgpAH and RgpB than rRgpB-PP. It was also found that both rRgpA-PP and rRgpB-PP weakly inhibited rKgp with IC_50_ of 13 ± 1.5 and 11 ± 2 µM, respectively (S11 Table). Similar to a previous report^13^ the rKgp-PP produced limited inhibition of rKgp with a *K*_*i*_ of 37.5 µM (95% CI of 36 to 39 µM) (S6 Fig) and showed no inhibition of rRgpAH or RgpB.

**Figure 4:**
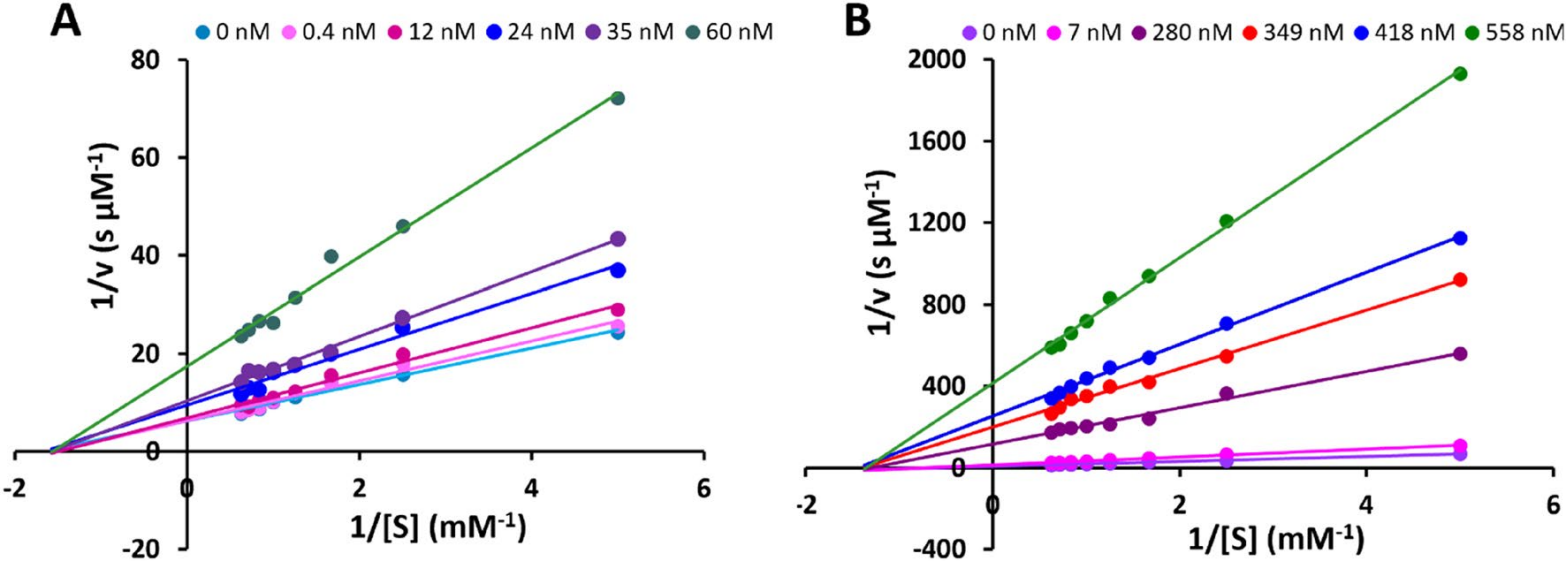
Lineweaver–Burk plot of the inhibition of rRgpAH. Inhibition by rRgpA-PP (**A**) and rRgpB-PP (**B**) using the substrate BApNA. Propeptide concentrations used are indicated in the key in each panel.

The type of rRgpA-PP and rRgpB-PP mediated inhibition of rRgpAH was determined using a Lineweaver–Burk plot. The addition of increasing concentrations of both rRgpA-PP and rRgpB-PP decreased the rate of product formation, while the *K*_m_ values remained the same (Fig. 4). This indicated non-competitive inhibition of rRgpAH by both rRgpA-PP and rRgpB-PP. Furthermore, the similar *K*_i_ values of rRgpA-PP and rRgpB-PP determined for different substrate concentrations indicated that the inhibition was substrate-independent (S12 Table).

## Discussion

In this study, *P. gingivalis* wild-type plus RgpB and RgpB-Kgp double mutants were transformed with a variety of suicide plasmids and each produced active, soluble recombinant RgpA_cat_ in the culture fluid. Furthermore, the results indicated that neither RgpB nor Kgp is necessary for the processing and release of the recombinant RgpAs from the cell surface. Rather, as yet unidentified *P. gingivalis* proteinases, or the recombinant RgpA, can participate in this process. However, the C-terminal peptide identified for rRgpA_cat_ produced by the RgpB-Kgp-negative strain ECR806 does not indicate rRgpA-mediated cleavage from the cell. Rather, an alternative Lys-specific enzyme to Kgp may be involved. Recently, the *P. gingivalis* lysyl endopeptidase PepK was identified^24^. PepK activity forms approximately one-fortieth of the Lys-specific activity of *P. gingivalis,* and processing of PepK by RgpA has been indicated^24^ suggesting a co-relationship of these enzymes on the cell surface. The role of PepK in *P. gingivalis* is unclear but it could be postulated that a potential function for PepK is to process *P. gingivalis* surface proteins. Thus, in the absence of Kgp activity PepK activity could provide one explanation for the release of rRgpA_cat_, rRgpAH, and rRgpATH from the surface of *P. gingivalis*. Notably, MS analysis revealed that purified rRgpAH was predominantly truncated with loss of the C-terminal KK residues. The carboxypeptidase CPG70 has been indicated to process proteinases and adhesins of RgpA and Kgp by removing Lys residues at the C-termini of the processed polypeptide fragments^25^. Thus, CPG70 activity could explain the missing Lys residues of rRgpAH.

Veillard et al.^19^ have purified a hexahistidine-tagged RgpB from *P. gingivalis* culture fluid and showed it was biochemically indistinguishable from the native RgpB. The fidelity of the rRgpAH enzyme produced here was evidenced by the kinetic relationship between rRgpAH and RgpB and similarity to HRgpA kinetics as determined in previous reports^13,15,19,26,27^. For example, it was observed that HRgpA activity increased 5 to sixfold in the presence of 100 mM glycylglycine^27^ which correlates with our observation of ~ sixfold increase in RgpAH activity in the presence of glycylglycine. Glycylglycine stimulated gingipain activity in the order rRgpAH > rKgp > RgpB with rRgpAH activity twofold higher than RgpB, a result consistent with a previous study^15^. Without glycylglycine, the kinetics of rRgpAH and purified RgpB were similar with values of K_m_ and *k*_cat_ that agree with values in the literature for HRgpA and RgpB^13,15,19,26,27^. This rRgpAH fidelity also extended to similar responses to inhibition by gingipain propeptides observed by others^20^. The results therefore also suggest that the presence of the adhesin domains does not significantly influence RgpA proteinase catalytic activity, at least when using BapNA as substrate. Overall, this indicates the potential usefulness of rRgpAH for improving our understanding of RgpA activity and function.

The *K*_m_ of purified rKgp for GPKNA was determined here to be 202 ± 18 µM when assayed in the presence of 180 mM of glycylglycine and fivefold less at 39 ± 2.4 µM in the absence of glycylglycine; thus, glycylglycine has a clear effect on the affinity of the enzyme for GPKNA. The *K*_m_ using GPKNA as substrate (in the absence of glycylglycine) reported for purified rKgp was 46 µM^13^ and 50 µM for the Kgp released into culture fluid by *P. gingivalis* HG66^28^ which is in agreement with the data in this study. Furthermore, the calculated rKgp *k*_cat_ in the absence of glycylglycine was 5.4 s^−1^_,_ which compared well with the *k*_cat_ value of 4.5 s^−1^ determined previously^13^ and five-fold less than the calculated *k*_*cat*_ in the presence of glycylglycine. Thus the activation of gingipains by glycylglycine is very closely correlated to the change of the *K*_m_^15,23^.

It was noted in a survey of the sequences of a panel of 13 *P. gingivalis* strains that the RgpA and RgpB catalytic domains were highly conserved across the strains but that the propeptides of RgpAs and RgpBs differed, with 43 substitutions and 3 indels across 199 residues^29^. It was suggested that the propeptides may have distinct binding sites on the enzymes. We determined a slightly lower *K*_i_ for rRgpA-PP than rRgpB-PP against both rRgpAH and RgpB. Others have also observed a lower, albeit small, difference in *K*_i_ of a recombinant RgpA propeptide against RgpA (as HRgpA) and RgpB relative to RgpB-propeptide^20^ This supports that the difference in *K*_i_ we measured was a subtle but real observation and supports a contention of difference in propeptide binding sites. A future experiment to explore the impact of this *K*_i_ difference could be temporal analysis of the maturation/activation of propeptide-proteinase chimeras. These chimeras could have RgpA propeptide preceding the RgpB catalytic domain or RgpB propeptide preceding the RgpA catalytic domain. Utilizing modifications to the pRgpAH suicide vector developed here, chimeric propeptide-RgpA/B and RgpB-His encoding genes could be produced from the same genetic background as rRgpAH, utilising the same promoter and ribosome binding sequences. Comparisons between the chimera rate of active enzyme production and rRgpAH and RgpB active enzyme production can then be made. The rKgp-PP was found to be a weak inhibitor against purified rKgp with *K*_i_ 38 µM, whereas both rRgpA-PP and rRgpB-PP were better, albeit still weak, inhibitors against rKgp with *K*_i_ 13 µM and 11 µM, respectively. These results are consistent with a previous report where the *K*_i_ value of Rgp propeptides determined against rKgp was 10 µM^20^. It is unclear why the RgpA propeptide is more effective at inhibiting RgpB and Kgp than either of the cognate propeptides of these enzymes.

## Conclusion

A cloning strategy was devised that enabled functional histidine-tagged RgpA_cat_, (rRgpAH), to be released into *P. gingivalis* culture fluid from where it was purified. Despite the high sequence similarity between the RgpA and RgpB catalytic domains, the small number of sequence differences between the two enzymes has resulted in some differences in specific activity.

## Methods

### Growth of *P. gingivalis* and production of *P. gingivalis* mutants

*P. gingivalis* were routinely grown at 37° C on 10% lysed, defibrinated, horse blood agar solid media and incubated in an anaerobic atmosphere (5% H_2_, 10% CO_2,_ and 85% N_2_). Liquid cultures were grown in brain–heart infusion broth supplemented with 5 µg mL^−1^ hemin, 5 µg mL^−1^ menadione, and 5 mg mL^−1^ cysteine. Agar plates and liquid media were also supplemented with 1 µg mL^−1^ tetracycline, 10 µg mL^−1^ erythromycin, or 1 µg mL^−1^ ampicillin antibiotics as appropriate. *P. gingivalis* mutants were produced by electroporation of *P. gingivalis* cells with linearized suicide plasmids, performed as previously described^30^. Selected clones were screened for the presence of the appropriate homologous recombination event by gDNA isolation (Dneasy Blood & Tissue Kits, QIAGEN, Germany) and PCR with the appropriate oligonucleotide primers (S1 Table).

*P. gingivalis* W50B, a mutant with *rgpB* inactivated by insertion of a *tetQ* cassette^10^ was transformed with a *kgp::cepA* cassette^31^ to inactivate *kgp*. Correct *cepA* integration into *kgp* in the ampicillin-resistant transformants was confirmed using PCR as previously described^31^. Inactivation of *rgpB* of ECR803 using an *rgpB*::*tetQ* suicide vector was conducted as described^10^.

*E. coli* were grown at 37° C using Luria Bertani (LB) broth and agar. *E. coli* α-Select gold efficiency commercial chemically competent cells (Bioline) were transformed with plasmid using the heat-shock method as per the manufacturer’s instructions. Transformed cells were plated on LB agar containing 100 µg mL^−1^ ampicillin and incubated overnight. The resulting transformants were screened for the presence of the appropriate insert using PCR followed by plasmid DNA isolation and sequencing with the appropriate nucleotide primers (S1 Table).

PCR for *E. coli* plasmid clone selection used BIOTAQ™ polymerase (Bioline) as per manufacturer instructions. PCR amplification of recombinant *P. gingivalis* genomic DNA (gDNA) used Crimson *Taq* polymerase (NEB). PCR products were purified using the QIAquick PCR purification kit, and gDNA was extracted from *P. gingivalis* using the Dneasy® Blood and Tissue kit (QIAGEN, Germany) as per the manufacturer’s instructions. Plasmid DNA was extracted from *E. coli* using the QIAprep® Spin Miniprep kit (QIAGEN). Sanger sequencing was performed at the Australian Genome Research Facility (AGRF, Melbourne).

The recombination cassette within the suicide plasmid pRgpArelease (6375 bp) was synthesized by Bioneer (Bioneer Pacific, Australia) and ligated into the Bioneer cloning vector pBHA. Before electroporation into *P. gingivalis,* pRgpArelease, and related suicide plasmids were linearized using BglI restriction endonuclease (NEB). Splicing by overlap extension PCR (SOE-PCR) was used to modify pRgpArelease to incorporate codons for GHHHHHHG or LPVRGSGHHHHHHG 5-prime to the codons for ^661^KK^662^, producing pRgpAH and pRgpATH, respectively. The primers used in each SOE-PCR are listed in the S1 Table. Initially, two PCR amplicons were prepared with pRgpArelease plasmid as the template. The two PCR amplicons were purified and spliced by the overlapping sequence using PCR with the external primers F01 and R01. The amplicons were digested using SnaBI and PstI-HF (Biolabs Inc. MA, USA) and ligated into SnaBI and PstI-HF digested pRgpArelease. The sequence of the SOE-PCR-derived inserts were verified by Sanger sequencing (AGRF, Melbourne).

### SDS-PAGE and western immunoassay

Protein samples were resolved via SDS-PAGE using 10% (w/v) or 4–12% (w/v) gradient NuPAGE™ Bis–Tris Protein Gels (Life Technologies Australia Pty Ltd) and stained using Simply Safe Stain (Invitrogen) according to the manufacturer protocol. To conduct western immunoassay proteins in SDS-PAGE gels were transferred to a polyvinylidene difluoride membrane (Life Technologies) using Bolt™ Transfer Buffer (Life Technologies). Membranes were blocked using 5% (w/v) skimmed milk powder in PBS buffer (137 mM NaCl, 10 mM NaH_2_PO_4_·H_2_O, 2.7 mM KCl, pH 7.4) for one hour. The anti-His-tag monoclonal antibody (Sigma-Aldrich) was diluted in PBST buffer (137 mM NaCl, 10 mM NaH_2_PO_4_, 2.7 mM KCl, with 0.2% Tween-20, pH 7.4) and used to probe the membrane overnight at 4º C in the same blocking solution. The blot was washed four times in 25 mL of PBST buffer for 10 min each then incubated at room temperature for 1 h with rabbit anti-mouse IgG horseradish peroxidase (HRP)-conjugated antibody (Sigma-Aldrich) in blocking solution. Following three washes in PBST for 15 min each, HRP activity was detected using Immobilon™ Western HRP chemiluminescent substrate with a luminol peroxidase solution according to the manufacturer’s instructions (Millipore Corporation, MA, USA) with image capture using a LAS 3000 Imager (Fujifilm, Japan).

### Production of rRgpAH

ECR832 was grown in supplemented BHI broth incubated anaerobically at 37° C for 72 h. After incubation, the culture broth was centrifuged at 8000 g for 30 min, and the supernatant was clarified by filtration using a 0.22 µM filter. To reduce autocatalytic degradation of rRgpAH, the pH of the clarified supernatant was reduced to pH 5.5 using glacial acetic acid before storage at 4º C. Immediately before affinity purification, the pH of the ECR832 clarified supernatant was raised to pH 7.0 using NaOH.

TALON® metal affinity resin (Co^2+^ pre-charged; Takara Bio Inc., Japan) was equilibrated by adding 10 bed volumes of equilibration buffer (50 mM NaH_2_PO_4_, 300 mM NaCl, pH 7.0). The filtered culture supernatant was incubated with the prepared resin at a sample-to-resin volume ratio of 100:1 for 1 h at 4° C with mixing. The protein-resin mixture was packed into an empty PD-10 column fitted with a frit (GE Healthcare, USA), and the flow-through was discarded. The column was washed five times with 2 resin bed volumes of wash buffer (50 mM NaH_2_PO_4_, 300 mM NaCl, 10 mM imidazole, pH 8.0). The bound proteins were eluted by 5 bed volumes of elution buffer (50 mM NaH_2_PO_4_, 300 mM NaCl, 150 mM imidazole, pH 7.0). The fractions containing the majority of His-tagged proteins determined by SDS-PAGE and Arg-proteinase assay were pooled and concentrated using a Vivaspin® 20 centrifugal filter device with a 5 kDa molecular weight cut-off. Retentates were desalted using a PD-10 column (GE Healthcare) equilibrated with 50 mM NaH_2_PO_4_, 300 mM NaCl, pH 7.0. Finally, the eluent from the PD-10 desalting column was subjected to gel-filtration (HiLoad 16/600 Superdex 75 prep grade prepacked column, GE Healthcare) with TBS buffer (50 mM Tris,150 mM NaCl, pH 7.5).

### Waters Vion IMS Q-TOF spectroscopy

Intact protein samples were analyzed using a Waters Acquity H-class UPLC coupled to a Vion IMS QTOF mass spectrometer (Milford, MA, USA). The LC system was equipped with a PLRP-S column (5 µm, 1000 Å, 2.1 × 50 mm, Agilent, Santa Clara, CA, USA). The eluents used for the LC were water with 0.1% v/v formic acid for solvent A and acetonitrile with 0.1% v/v formic acid for solvent B. Typically, for each LC–MS experiment, 1 µg of the intact protein was loaded onto the column at 200 µL min^−1^ using 1% B for 2 min. The gradient used was from 1% B to 95% B for 8 min, 95% B to 95% B in 2 min, and 95% B to 1% B in 0.5 min followed by column washing and re-equilibration. The MS experiments were performed using an electrospray ionization source in positive mode. The capillary spray voltage, source temperature, desolvation temperature, cone gas flow, desolvation gas flow, and collision energy were set to 2.75 kV, 100 °C, 250 °C, 50 L h^−1^, 600 L h^−1^, and 15 eV. Full scan MS spectra had an m/z range of 100–4000. Mass spectrometry data were deconvoluted using UNIFI software with the MaxEnt1 algorithm (Waters, Milford, MA, USA).

### In-gel trypsin digestion of protein for use in MS

In-gel digestion and LC–MS/MS were performed essentially, as previously described^32^. For in-solution digestion of protein, approximately 20 µg of protein was added in 25 µL of 50 mM tetraethylammonium tetra-hydroborate (TEAB) buffer to a 1.5 mL tube. Trifluoroethanol denaturation agent (25 μL) was added and 1 µL of 500 mM tris(2-carboxyethyl) phosphine (TCEP) then the sample was homogeneously mixed by vortexing. The protein was denatured by heating at 60° C for 45 min, after which 20 μL of 100 mM iodoacetamide (IAM) in 50 mM TEAB buffer stock solution was added, and the solution vortexed briefly. The solution was kept at room temperature for 1 h in the dark. The excess IAM was neutralized by adding 1 µL of 500 mM TCEP, then 300 μL of water was added to dilute denaturant, and the pH was raised by adding 100 μL of 50 mM TEAB.

### Mass spectrometry for peptide analysis

The MS experiments were performed using a nanoelectrospray ionization source at positive mode and a Fusion Lumos Orbitrap mass spectrometer (Thermo Fisher Scientific, San Jose, CA). The spray voltages, capillary temperature, and S-lens RF level were set to 1.9 kV, 275° C, and 30%. The mass spectrometry data was acquired with a 3-s cycle time for one full scan of MS spectra and as many data-dependent higher-energy collisional dissociation (HCD)-MS/MS spectra as possible. Full scan MS spectra had an m/z range of 400–1500, a resolution of 120,000 at m/z 200, an auto gain control (AGC) target value of 4e5, and a maximum ion trapping time of 50 ms. The data-dependent HCD-MS/MS of peptide ions (charge states from 2 to 5) was performed using an m/z isolation window of 1.6, an AGC target value of 5e4, normalized collision energy (NCE) of 35%, a resolution of 15,000 at m/z 200 with a maximum ion trapping time of 54 ms. All mass spectrometry data were acquired using an Orbitrap mass analyzer. Dynamic exclusion was used for 30 s.

### Proteinase assay

Arg- and Lys-gingipain proteolytic activities were determined at 37° C using BAapNA or GPKNA respectively in a final volume of 200 µL, with Tris-buffer (50 mM Tris, 150 mM NaCl, 5 mM CaCl_2_, pH 8.0), in 96 well flat-bottom plates. l-Cysteine hydrochloride was added from a freshly made 1 M stock adjusted to pH 7.0, and glycylglycine was added from a 1.5 M stock, pH 7.0. Purified rRgpAH, RgpB, and rKgp were used at 5 nM. Release of *p*–nitroaniline was determined by change in absorbance at 405 nm, at 1 s intervals, for up to 99 repeats, using a Victor^3^ 1420 Multilabel Counter (Perkin Elmer, MA, USA).

### Kinetic determinations

To calculate the *V*_max_ and *K*_m_, the initial velocity region of a series of enzymatic reactions in the presence of increasing concentrations of the substrate was determined. The curves were fitted individually by non-linear regression analysis to the Michaelis–Menten expression (v = *V*_max_[S]/(*K*_m_ + [S]), where v is the rate of reaction, *V*_max_ is the maximum rate, [S] is the concentration of substrate and *K*_m_ is the half of *V*_max_^33^ using the program GraphPad Prism (GraphPad Software, Inc.). The rate of product formation was expressed in nM s^−1^, calculated with the extinction coefficient of *p*-nitroaniline being 9960 M^−1^ cm^−1^ at 405 nm and a path length of 0.6 cm. The *V*_max_ was calculated using the Michaelis–Menten equation (*V*_max =_ *vK*_m_/[S] + *v*). The *V*_max_ of the purified enzyme was also calculated using the equation *V*_max =_ *k*_cat_/[E_total_], where [E]_0_ is the concentration of enzyme, and *k*_cat_ is the turnover number. The turnover number of the purified rRgpAH, RgpB, and rKgp were calculated using the equation *k*_cat =_ *V*_max_/[E_total_]. The catalytic efficiency was calculated using the *k*_cat_/*K*_m_ ratio. The *K*_m_ and *V*_max_ were also calculated according to Lineweaver–Burk double reciprocal plot using the Eq. (1/*v* = 1/*V*_max_ + *K*_m_/*V*_max_ × 1/[S]).

### Protease inhibition assays

To conduct propeptide-proteinase inhibition analyses recombinant proteins of the gingipain propeptides (rRgpA-PP, rRgpB-PP, rKgp-PP) were purified from *E. coli* cell lysates using a combination of His-trap nickel-affinity purification and size exclusion chromatography as previously described^13^. The rRgpA-PP production used the same vector system rRgpB-PP and rKgp-PP vector systems^13^ except there was substitution of the *rgpA* codons for the RgpA propeptide in the expression vector. The *rgpA* propeptide codons were amplified using PCR, the oligonucleotide primers RgpA-PP-for plus RgpA-PP-rev (S1 Table), and *P. gingivalis* W50 DNA as a template.

Inhibition assays were performed in a total volume of 200 µL in a 96-well microtiter plate. Appropriate concentrations of rRgpA-PP, rRgpB-PP, or rKgp-PP were added to the protease assay prior to the addition of the substrate. To determine the type of inhibition, increasing concentrations of rRgpA-PP or rRgpB-PP were added to purified rRgpAH or RgpB separately and incubated for 15 min at 37° C in assay buffer supplemented with 20 mM l-cysteine hydrochloride, and 300 mM glycylglycine pH 7.0, after which substrate was added at several concentrations ranging from 0.2 mM to 1.6 mM. The plates were immediately placed in the Victor^3^ 1420 Multilabel Counter (Perkin Elmer, MA, USA) and the change in absorbance at 405 nm was monitored at 10 s intervals. The IC_50_ were calculated using four-parameter logistic non-linear regression models (Y = Bottom + (Top–Bottom)/(1 + 10^((LogIC_50_-X) *Hillslope)) in GraphPad Prism 7.

The initial rates were plotted using a Lineweaver–Burk plot (1/v plotted against 1/[S]_0_ giving intercepts at 1* V*_max_ and − 1/*K*_m_). The *K*_i_ were calculated for different substrate concentrations. Inhibition modelling (*V*_maxinh_ = *V*_max_/(1 + I/*K*_i_), Y = *V*_max_ X/(*K*_m_ + X)) was performed in the GraphPad PRISM software. Each rate of reaction in the presence of inhibitors was determined by subtracting rates of control wells.

### Statistical analysis

Statistical analysis was done using Graph Pad Prism 7 software. The data were analyzed with two-way ANOVA to determine statistical significance. Values are expressed as the ± standard error of the mean or at a 95% confidence interval. *p* < 0.05 was considered statistically significant.

## Supplementary Information

Supplementary Information.

## Data Availability

Data forming this report are available within this manuscript and in supporting information. DNA sequence information can be found under GenBank Accession numbers OP456363, OP456364, and OP456365. The mass spectrometry proteomics data have been deposited to the ProteomeXchange Consortium via the PRIDE^34^ partner repository and can be accessed via the link http://www.ebi.ac.uk/pride/archive/projects/PXD036859.
