# Supplementary material for: Production and properties of adhesin-free gingipain proteinase RgpA
Source: Sci Rep. 2023 Jul 4;13:10780. doi: 10.1038/s41598-023-37534-x (PMC10319874; doi:10.1038/s41598-023-37534-x)
Supplement: Supplementary file 1 — Supplementary Information. [file 41598_2023_37534_MOESM1_ESM.pdf]

Production and properties of adhesin-free gingipain proteinase RgpA

Abu Sayeed M. Mahmud, Christine A. Seers, N. Laila Huq, Lianyi Zhang, Catherine A. Butler, Keith J. Cross, and Eric C. Reynolds*.

SUPPORTING INFORMATION

S1 Table: Oligonucleotides used in this study

S2 Table: List of peptides derived from the LCMS analysis of tryptic digested SDS-PAGE gel band (a) of ECR803 day 1 VFSN proteins presented in Fig. 2.

S3 Table: List of peptides derived from the LCMS analysis of tryptic digested SDS-PAGE gel band (b) of ECR803 day 1 VFSN proteins presented in Fig. 2.

S4 Table: List of peptides derived from the LCMS analysis of tryptic digested SDS-PAGE gel band (c) of ECR804 day 1 VFSN proteins presented in Fig. 2.

S5 Table: List of peptides derived from the LCMS analysis of tryptic digested SDS-PAGE gel band (e) of ECR805 day 1 VFSN proteins presented in Fig. 2.

S6 Table: List of peptides derived from the LCMS analysis of tryptic digested SDS-PAGE gel band (d) of ECR804 day 1 VFSN proteins presented in Fig. 2.

S7 Table: List of peptides derived from the LCMS analysis of tryptic digested SDS-PAGE gel band (f) of ECR805 day 1 VFSN proteins presented in Fig. 2.

S8 Table: List of peptides derived from the LCMS analysis of tryptic digested SDS-PAGE gel band (g) of ECR806 day 1 VFSN proteins presented in Fig. 2.

S9 Table: List of peptides derived from the LCMS analysis of tryptic digested SDS-PAGE gel band (h) of ECR806 day 1 VFSN proteins presented in Fig. 2.

S10 Table: Peptides derived from the LC-MS analysis of trypsin digested purified rRgpAH.

S11 Table: The Ki of rRgpA-PP, rRgpB-PP, and rKgp-PP against purified gingipains.

S12 Table: The K_i of rRgpA-PP and rRgpB-PP against rRgpAH.

S1 Fig: PCR analysis of transformed *P. gingivalis* genomic DNA

S2 Fig: LC-MS analysis of the rRgpA secreted by ECR806.

S3 Fig: The LC-MS analysis of trypsin digested peptides of rRgpATH in the ECR821 VFSN band j (Fig 2.).

S4 Fig: The LC-MS analysis of tryptic digested rRgpAH peptides.

S5 Fig: SDS-PAGE and MS analysis of purified rRgpAH.

S6 Fig: The kinetics of rKgp using substrate GPKNA.

S7 Fig: Original gel and blot images

S1 Table: Oligonucleotides used in this study

Name	Sequence	Target DNA	Purpose
**RgpAhr3pR**	ACAAAAATGGAGCTGTGCCG	3' to *rgpA*	Genome integration verification, *rgpA* locus
**pgmAR1**	AAAAGGCAGGAGTTGTTTTGC GT	5' to *rgpA*	Genome integration verification, *rgpA* locus
**R01**	GCTGACCCGTAAAACGAACG	*ermF* at 5', reverse; pRgpArelease	Genome integration verification, *rgpA* locus, and SOE PCR to produce *rgpAH/rgpATH*
**F01**	GTTCGGTTCTGCAGTTGTCG	pRgpArelease	SOE PCR to produce *rgpAH/rgpATH*
**R02**	ACCATGATGATGATGATGATG ACCTGAGCCTCTAGGGACCAG CGTGCTATTAGTGTTGAT	pRgpArelease	SOE PCR to produce *rgpATH.* Anneals TINTNS codons
**F02**	CTGGTCCCTAGAGGCTCAGGT CATCATCATCATCATCATGGT AAGAAGGCAAGCTATACC	pRgpArelease	SOE PCR to produce *rgpATH*
**R03**	ACCATGATGATGATGATGATG ACCCGTGCTATTAGTGTTGAT GG	pRgpArelease	SOE PCR to produce *rgpAH*. Anneals TINTNS codons
**F03**	GGTCATCATCATCATCATCAT GGTAAGAAGGCAAGCTATAC CTA	pRgpArelease	SOE PCR to produce *rgpAH*
**tetQ5prev**	GGCATTTGAGTATTTCCCATTC G	*tetQ*	Genome integration verification, *rgpB* locus
**E72s-1F**	CGAGGCAAGTCCGGATG	*rgpB*	Genome integration verification, *rgpB* locus
**RgpA-PP-for**	ACGCAGCATATGCAGCAGAC AGAGTTGGGACGCA	*rgpA*	Production of rRgpA-PP
**RgpA-PP-rev**	CTCTCTCTCGAGTTAACGCCCT GGCTCGTAGTTCATG	*rgpA*	Production of rRgpA-PP

S2 Table: List of peptides derived from the LCMS analysis of tryptic digested SDS-PAGE gel band (a) of ECR803, D1, VFSN proteins presented in Fig. 2.

Peptides	MaxQuant Score	Theoretical neutral monoisotopic mass	Mass Error (ppm)
^{64}GIGQVPTYTEGVNLSEK^{80}	15	1790.9	1.121
^{99}EMKVEVVSSK^{108}	74.9	1134.59	-0.172
^{102}VEVVSSKFIEKK^{113}	51.2	1391.8	0.27
^{109}FIEKKNVLIAPSK^{113}	72	1485.89	0.184
^{114}KNVLIAPSK^{121}	146	968.6	-0.178
^{115}NVLIAPSK^{121}	54	840.5	0.075
^{115}NVLIAPSKGMIMR^{126}	86	1428.79	-0.109
^{122}GMIMRNEDPK^{131}	27.5	1189.55	0.177
^{132}KIPYVYGK^{139}	62.8	966.55	0.147
^{133}IPYVYGK^{139}	67	838.45	0.028
^{201}NILNKK^{206}	54	728.45	0.289
^{206}KGTFAGFEDTYK^{217}	153	1362.64	0.0029
^{207}GTFAGFEDTYK^{217}	129.8	1234.55	0.042
^{207}GTFAGFEDTYKR^{218}	155.5	1390.65	-0.297
^{228}YTPVEEK^{234}	28	864.4	0.062
^{228}YTPVEEKQNGR^{238}	76	1319.64	0.042
^{239}MIVIVAK^{245}	106	772.48	0.421
^{239}MIVIVAKK^{246}	98	900.58	0.265
^{269}VAEDIASPVTANAIQQFVK^{287}	38	2000.05	-1.270
^{288}QEYEKEGNDLTYVLLIGDHK^{307}	124	2363.16	0.105
^{288}QEYEKEGNDLTYVLLIGDHKDIPAK^{312}	88.5	2887.46	-0.064
^{293}EGNDLTYVLLIGDHK^{307}	303	1685.86	-0.161
^{293}EGNDLTYVLLIGDHKDIPAK^{312}	239	2210.15	-0.229
^{308}DIPAKITPGIK^{318}	80	1151.69	-0.065
^{319}SDQVYGQIVGNDHYNEVFIGR^{339}	189.8	2409.13	0.362
^{340}FSCESKEDLKTQIDR^{354}	175.3	1797.85	0.134
^{340}FSCESKEDLK^{349}	63	1184.53	0.226
^{355}TIHYERNITTEDK^{367}	35.6	1618.79	-0.322
^{409}IIKCYDPGVTPK^{420}	78	1332.71	0.149
^{421}NIIDAFNGGISLANYTGHGSETAWGTSHFGTTHVK^{455}	8	3659.73	-2.166
^{490}AQKDGKPTGTVAIIASTINQSWASPMR^{516}	91.7	2827.46	0.001
^{517}GQDEMNEILCEKHPNNIK^{536}	53.7	2110.97	0.124
^{517}GQDEMNEILCEKHPNNIKR^{537}	91.5	2267.07	-0.251
^{537}RTFGGVTMNGMFAMVEK^{551}	106.8	1874.88	0.137
^{538}TFGGVTMNGMFAMVEK^{551}	213	1718.78	-0.433
^{552}YKKDGEK^{558}	40	866.44	0.761
^{555}DGEKMLDTWTVFGDPSLLVR^{574}	107	2278.13	-0.432
^{575}TLVPTK^{580}	55	657.4	-0.026
^{649}ETVIKTINTNSTK^{661}	113.5	1447.78	0.087

S3 Table: List of peptides derived from the LCMS analysis of tryptic digested SDS-PAGE gel band (b) of ECR803, D3, VFSN proteins presented in Fig. 2.

Peptides	MaxQuant Score	Theoretical neutral monoisotopic mass	Mass Error (ppm)
^{228}YTPVEEK^{234}	65.4	864.66	0.249
^{228}YTPVEEKQNGR^{238}	74	1319.64	0.035
^{239}MIVIVAK^{245}	112	772.48	0.143
^{239}MIVIVAKK^{246}	111.6	900.58	0.265
^{239}MIVIVAKKYEGDIK^{252}	14	1605.9	0.141
^{239}MIVIVAKKYEGDIKDFVDWK^{258}	38.5	2396.28	0.100
^{269}VAEDIASPVTANAIQQFVK^{287}	39	2000.05	0.013
^{288}QEYEKEGNDLTYVLLIGDHK^{307}	90.8	2363.16	0.519
^{288}QEYEKEGNDLTYVLLIGDHKDIPAK^{312}	73.6	2887.46	-0.303
^{293}EGNDLTYVLLIGDHKDIPAKITPGIK^{318}	25	2819.54	-0.024
^{293}EGNDLTYVLLIGDHKDIPAK^{312}	183.2	2210.15	-0.447
^{293}EGNDLTYVLLIGDHK^{307}	242	1685.86	-0.407
^{308}DIPAKITPGIK^{318}	103.4	1151.69	0.034
^{319}SDQVYGQIVGNDHYNEVFIGR^{339}	181.5	2409.13	-0.416
^{340}FSCESKEDLK^{349}	172.6	1184.53	-0.142
^{340}FSCESKEDLKTQIDR^{354}	139.8	1797.85	-0.416
^{350}TQIDRTIHYER^{360}	94.8	1430.72	-0.154
^{355}TIHYER^{360}	61	817.40	-0.325
^{355}TIHYERNITTEDK^{367}	75.8	1618.79	-0.035
^{409}IIKCYDPGVTPK420	95.4	1332.71	0.0192
^{421}NIIDAFNGGISLANYTGHGSETAWGTSHFGTTHVK^{455}	20	3659.73	-0.201
^{490}AQKDGKPTGTVAIIASTINQSWASPMR^{516}	65	2827.46	-0.534
^{517}GQDEMNEILCEKHPNNIK^{536}	165.4	2110.97	0.178
^{517}GQDEMNEILCEKHPNNIKR^{537}	60	2267.07	-0.551
^{537}RTFGGVTMNGMFAMVEK^{551}	182	1874.88	-2.48
^{538}TFGGVTMNGMFAMVEKYKK^{554}	46	2138.03	0.107
^{552}YKKDGEK^{558}	38.3	866.44	0.116
^{555}DGEKMLDTWTVFGDPSLLVR^{574}	99	2278.13	-0.036
^{575}TLVPTK^{580}	53	657.4	0.074
^{615}MFGSAVVENGTATINLTGLTNESTLTLTVVGYNKETVIK^{653}	74.5	4085.12	-2.496
^{649}ETVIKTINTNSTK^{661}	236.4	1447.78	0.156

S4 Table: List of peptides derived from the LCMS analysis of tryptic digested SDS-PAGE gel band (c) from ECR804, D1, VFSN proteins as shown in Fig. 2.

Peptides	MaxQuant Score	Theoretical neutral monoisotopic mass	Mass Error (ppm)
^{56}FTEVQTPK^{63}	125	948.49	-0.049
^{66}GIGQVPTYTEGVNLSEK^{80}	89	1790.9	0.310
^{99}EMKVEVVSSK^{108}	153.54	1134.59	-0.236
^{102}VEVVSSKFIEKK^{113}	127.56	1263.7	-0.590
^{109}FIEKKNVLIAPSK^{113}	31.7	1485.89	0.116
^{114}KNVLIAPSK^{121}	123	968.6	-0.052
^{114}KNVLIAPSKGMIMR^{126}	68	1556.88	-0.067
^{115}NVLIAPSK^{121}	73	840.5	0.122
^{115}NVLIAPSKGMIMR^{126}	117	1428.79	-0.230
^{122}GMIMRNEDPK^{131}	44.4	1189.55	-0.132
^{132}KIPYVYGK^{139}	84.7	966.55	0.001
^{133}IPYVYGK^{139}	96	838.45	0.043
^{143}FFPGEIATLDDPFILRDVR^{161}	63	2220.15	2.114
^{162}GQVVNFAPLQYNPVTKTLR^{180}	131.3	2144.17	-0.101
^{201}NILNKKGTFAGFEDTYKR^{218}	38	2101.09	0.256
^{206}KGTFAGFEDTYK^{217}	110.3	1362.64	-0.152
^{207}GTFAGFEDTYK^{217}	138.3	1234.6	0.204
^{207}GTFAGFEDTYKR^{218}	174	1390.65	0.045
^{228}YTPVEEK^{234}	37	864.4	0.003
^{228}YTPVEEKQNGR^{238}	55	1319.6	0.083
^{239}MIVIVAK^{245}	77.4	772.48	0.120
^{239}MIVIVAKK^{246}	99.4	900.58	0.138
^{288}QEYEKEGNDLTYVLLIGDHK^{307}	107.6	2363.16	0.079
^{288}QEYEKEGNDLTYVLLIGDHKDIPAK^{312}	45	2887.46	-0.171
^{293}EGNDLTYVLLIGDHK^{307}	201	1685.86	-0.019
^{293}EGNDLTYVLLIGDHKDIPAK^{312}	257	2210.15	-0.120
^{308}DIPAKITPGIK^{318}	83	1151.69	0.037
^{319}SDQVYGQIVGNDHYNEVFIGR^{339}	131.5	2409.13	-0.169
^{340}FSCESKEDLK^{349}	77	1184.53	0.004
^{340}FSCESKEDLKTQIDR^{354}	24.2	1797.85	-0.376
^{355}TIHYERNITTEDK^{367}	47	1618.79	-0.301
^{409}IIKCYDPGVTPK^{420}	67	1332.71	0.020
^{490}AQKDGKPTGTVAIIASTINQSWASPMR^{516}	103.4	2827.46	-0.204
^{517}GQDEMNEILCEKHPNNIKR^{537}	33	2267.07	0.172
^{538}TFGGVTMNGMFAMVEK^{551}	105	1718.78	-0.644
^{552}YKKDGEK^{558}	69.2	866.44	0.314
^{575}TLVPTK^{580}	53	657.4	0.188
^{615}MFGSAVVENGTATINLTGLTNESTLTLTVVGYNKETVIK^{653}	28.3	4085.1	-1.460
^{649}ETVIKTINTNSTK^{661}	188	1447.8	0.055

S5 Table: List of peptides derived from the LCMS analysis of tryptic digested SDS-PAGE gel band (e) from ECR805, D1, VFSN proteins as shown in Fig. 2.

Peptides	MaxQuant Score	Theoretical neutral monoisotopic mass	Mass Error (ppm)
^{56}FTEVQTPK^{63}	107	948.49	-0.075
^{66}GIGQVPTYTEGVNLSEK^{80}	47.8	1790.9	0.572
^{99}EMKVEVVSSK^{108}	64	1134.59	0.026
^{99}EMKVEVVSSKFIEKK^{113}	45.3	1651.88	-0.10
^{109}FIEKKNVLIAPSK^{113}	57	1485.89	0.06
^{114}KNVLIAPSK^{121}	157.8	968.6	-0.139
^{115}NVLIAPSK^{121}	60	840.5	-0.061
^{132}KIPYVYGK^{139}	81	966.55	-0.120
^{206}KGTFAGFEDTYK^{217}	157.5	1362.6	-0.181
^{207}GTFAGFEDTYK^{217}	74.6	1234.6	-0.094
^{207}GTFAGFEDTYKR^{218}	75	1390.65	-0.222
^{228}YTPVEEK^{234}	36.7	864.4	0.274
^{288}QEYEKEGNDLTYVLLIGDHK^{307}	58.7	2363.16	-0.063
^{288}QEYEKEGNDLTYVLLIGDHKDIPAK^{312}	57	2887.46	0.149
^{293}EGNDLTYVLLIGDHK^{307}	225.6	1685.86	0.117
^{293}EGNDLTYVLLIGDHKDIPAK^{312}	39	2210.15	-0.325
^{308}DIPAKITPGIK^{318}	42.4	1151.69	-0.116
^{319}SDQVYGQIVGNDHYNEVFIGR^{339}	110.6	2409.13	-0.282
^{340}FSCESKEDLK^{349}	64	1184.53	-0.187
^{421}NIIDAFNGGISLANYTGHGSETAWGTSHFGTTHVK^{455}	74.5	3659.73	-2.93
^{490}AQKDGKPTGTVAIIASTINQSWASPMR^{516}	24	2827.46	-0.333
^{538}TFGGVTMNGMFAMVEK^{551}	231	1718.78	-0.390
^{575}TLVPTK^{580}	65.9	657.4	0.044
^{615}MFGSAVVENGTATINLTGLTNESTLTLTVVGYNKETVIK^{653}	42.3	4085.1	-1.672
^{649}ETVIKTINTNSTK^{661}	134	1447.78	-0.012

S6 Table: List of peptides derived from the LCMS analysis of tryptic digested SDS-PAGE gel band (d) from ECR804, D3, VFSN proteins as shown in Fig. 2.

Peptides	MaxQuant Score	Theoretical neutral monoisotopic mass	Mass Error (ppm)
^{228}YTPVEEK^{234}	49	864.4	0.120
^{228}YTPVEEKQNGR^{238}	49	1319.6	0.086
^{239}MIVIVAK^{245}	103.3	772.48	0.133
^{239}MIVIVAKK^{246}	66	900.58	0.163
^{239}MIVIVAKKYEGDIK^{252}	42	1605.9	-0.436
^{239}MIVIVAKKYEGDIKDFVDWK^{258}	28	2396.28	0.138
^{288}QEYEKEGNDLTYVLLIGDHK^{307}	157.7	2363.16	0.292
^{288}QEYEKEGNDLTYVLLIGDHKDIPAK^{312}	50	2887.46	-0.029
^{293}EGNDLTYVLLIGDHK^{307}	277	1685.86	-0.271
^{293}EGNDLTYVLLIGDHKDIPAK^{312}	171.4	2210.15	-0.216
^{293}EGNDLTYVLLIGDHKDIPAKITPGIK^{318}	72	2819.54	0.123
^{308}DIPAKITPGIK^{318}	89.3	1151.69	-0.158
^{319}SDQVYGQIVGNDHYNEVFIGR^{339}	196.8	2409.13	-0.134
^{340}FSCESKEDLK^{349}	161	1184.53	0.034
^{340}FSCESKEDLKTQIDR^{354}	136.6	1797.85	-0.142
^{355}TIHYER^{360}	72.2	817.4	-0.233
^{355}TIHYERNITTEDK^{367}	45.3	1618.79	0.066
^{409}IIKCYDPGVTPK^{420}	86	1332.71	0.001
^{490}AQKDGKPTGTVAIIASTINQSWASPMR^{516}	97.8	2827.46	-0.547
^{517}GQDEMNEILCEKHPNNIK^{536}	41	2110.97	-0.116
^{517}GQDEMNEILCEKHPNNIKR^{537}	64.5	2267.07	-0.197
^{537}RTFGGVTMNGMFAMVEK^{551}	104.6	1874.88	0.453
^{538}TFGGVTMNGMFAMVEK^{551}	223	1718.78	-1.490
^{538}TFGGVTMNGMFAMVEKYKK^{554}	48.4	2138.03	0.567
^{552}YKKDGEK^{558}	55.3	866.44	0.261
^{555}DGEKMLDTWTVFGDPSLLVR^{574}	99.7	2278.13	-0.102
^{575}TLVPTK^{580}	63.6	657.4	0.001
^{649}ETVIKTINTNSTK^{661}	153.3	1447.78	0.182

S7 Table: List of peptides derived from the LCMS analysis of tryptic digested SDS-PAGE gel band (f) from ECR805, D3, VFSN proteins as shown in Fig. 2.

Peptides	MaxQuant Score	Theoretical neutral monoisotopic mass	Mass Error (ppm)
^{228}YTPVEEK^{234}	39	864.4	0.104
^{228}YTPVEEKQNGR^{238}	153	1319.6	-0.317
^{239}MIVIVAK^{245}	97.6	772.48	0.097
^{239}MIVIVAKK^{246}	128.6	900.58	-0.044
^{228}YTPVEEKQNGRMIVIVAK^{245}	40	2074.12	-0.160
^{239}MIVIVAKKYEGDIKDFVDWK^{258}	34	2396.28	0.214
^{265}TEVKVAEDIASPVTANAIQQFVKQEYEK^{292}	150	3134.6	0.017
^{269}VAEDIASPVTANAIQQFVK^{287}	176	2000.05	0.013
^{288}QEYEKEGNDLTYVLLIGDHK^{307}	56	2363.16	0.291
^{288}QEYEKEGNDLTYVLLIGDHKDIPAK^{312}	83	2887.46	0.091
^{293}EGNDLTYVLLIGDHK^{307}	86.7	1685.86	0.009
^{293}EGNDLTYVLLIGDHKDIPAK^{312}	195.3	2210.15	0.698
^{293}EGNDLTYVLLIGDHKDIPAKITPGIK^{318}	17	2819.54	0.430
^{308}DIPAKITPGIK^{318}	71.2	1151.69	0.034
^{308}DIPAKITPGIKSDQVYGQIVGNDHYNEVFIGR^{339}	45	3542.8	0.001
^{319}SDQVYGQIVGNDHYNEVFIGR^{339}	196.8	2409.13	-0.323
^{319}SDQVYGQIVGNDHYNEVFIGRFSCESKEDLKTQIDR^{354}	54	4188.98	0.149
^{340}FSCESKEDLK^{349}	169.3	1184.53	-0.183
^{340}FSCESKEDLKTQIDR^{354}	231.6	1797.85	0.0258
^{340}FSCESKEDLKTQIDRTIHYER^{360}	40	2597.25	-0.513
^{346}EDLKTQIDRTIHYER^{360}	78	1915.97	-0.037
^{350}TQIDRTIHYER^{360}	99.3	1430.72	-0.158
^{355}TIHYER^{360}	50	817.4	0.264
^{409}IIKCYDPGVTPK^{420}	127.2	1332.71	-0.025
^{421}NIIDAFNGGISLANYTGHGSETAWGTSHFGTTHVK^{455}	17	3659.73	-1.910
^{490}AQKDGKPTGTVAIIASTINQSWASPMR^{516}	112.8	2827.46	-0.392
^{517}GQDEMNEILCEKHPNNIK^{536}	47	2110.97	-0.214
^{517}GQDEMNEILCEKHPNNIKR^{537}	98	2267.07	-0.167
^{537}RTFGGVTMNGMFAMVEK^{551}	134	1874.88	-0.260
^{538}TFGGVTMNGMFAMVEK^{551}	292	1718.78	-0.132
^{538}TFGGVTMNGMFAMVEKYK^{553}	85.5	2009.94	-1.117
^{538}TFGGVTMNGMFAMVEKYKK^{554}	55	2138.03	-0.203
^{552}YKKDGEK^{558}	77.3	866.44	0.192
^{552}YKKDGEKMLDTWTVFGDPSLLVR^{574}	85.7	2697.38	-0.310
^{554}KDGEKMLDTWTVFGDPSLLVR^{574}	65.4	2406.2	-0.890
^{555}DGEKMLDTWTVFGDPSLLVR^{574}	158	2278.13	-0.310
^{575}TLVPTK^{580}	56.7	657.4	-3.820
^{649}ETVIKTINTNSTK^{661}	228.4	1447.78	0.053

S8 Table: List of peptides derived from the LCMS analysis of tryptic digested SDS-PAGE gel band (g) from ECR806, D1, VFSN proteins as shown in Fig. 2.

Peptides	MaxQuant Score	Theoretical neutral monoisotopic mass	Mass Error (ppm)
^{56}FTEVQTPK^{63}	153	948.49	0.064
^{56}FTEVQTPKGIGQVPTYTEGVNLSEK^{80}	91.8	2721.38	0.178
^{66}GIGQVPTYTEGVNLSEK^{80}	148.3	1790.9	-0.032
^{99}EMKVEVVSSK^{108}	153.64	1134.59	-0.144
^{102}VEVVSSKFIEK^{112}	90	1263.7	-0.22
^{102}VEVVSSKFIEKK^{113}	31	1391.8	-0.075
^{109}FIEKKNVLIAPSK^{113}	67	1485.89	0.153
^{114}KNVLIAPSK^{121}	158	968.6	-0.057
^{115}NVLIAPSK^{121}	56.4	840.5	0.281
^{115}NVLIAPSKGMIMR^{126}	53	1428.79	-0.143
^{122}GMIMRNEDPK^{131}	16	1189.55	0.133
^{132}KIPYVYGK^{139}	109	966.55	0.229
^{132}KIPYVYGKTYSQNK^{145}	58.8	1687.89	-0.254
^{133}IPYVYGK^{140}	75	438.45	-0.015
^{201}NILNKK^{206}	60.4	728.45	0.539
^{201}NILNKKGTFAGFEDTYKR^{218}	14	2101.09	-1.47
^{206}KGTFAGFEDTYK^{217}	153.3	1362.6	-0.106
^{207}GTFAGFEDTYK^{217}	113	1234.6	-0.195
^{207}GTFAGFEDTYKR^{218}	216	1390.65	-0.344
^{228}YTPVEEK^{234}	23	864.4	0.130
^{228}YTPVEEKQNGR^{238}	31	1319.6	-0.177
^{239}MIVIVAK^{245}	92.7	772.48	0.215
^{246}KYEGDIKDFVDWK^{258}	234	1641.8	-0.399
^{253}DFVDWK^{258}	95.8	808.37	-0.382
^{269}VAEDIASPVTANAIQQFVK^{287}	243.4	2000.05	0.385
^{269}VAEDIASPVTANAIQQFVKQEYEK^{292}	42	2677.35	-1.062
^{288}QEYEKEGNDLTYVLLIGDHK^{307}	69.4	2363.16	0.504
^{288}QEYEKEGNDLTYVLLIGDHKDIPAK^{312}	36.2	2887.46	-0.372
^{293}EGNDLTYVLLIGDHK^{307}	241	1685.86	0.0124
^{293}EGNDLTYVLLIGDHKDIPAK^{312}	69.5	2210.15	-0.747
^{308}DIPAKITPGIK^{318}	77.6	1151.69	0.286
^{319}SDQVYGQIVGNDHYNEVFIGR^{339}	75.6	2409.13	-0.097
^{340}FSCESKEDLKTQIDR^{354}	31	1797.85	-0.057
^{412}CYDPGVTPK^{420}	66.3	978.44	-0.063
^{421}NIIDAFNGGISLANYTGHGSETAWGTSHFGTTHVK^{455}	97.4	3659.73	-3.570
^{490}AQKDGKPTGTVAIIASTINQSWASPMR^{516}	81.7	2827.46	-0.274
^{538}TFGGVTMNGMFAMVEK^{551}	214	1718.78	-0.638
^{552}YKKDGEK^{558}	67.5	866.44	0.575
^{552}YKKDGEKMLDTWTVFGDPSLLVR^{574}	51	2697.38	0.100
^{575}TLVPTK^{580}	38.6	657.4	0.126
^{649}ETVIKTINTNSTK^{661}	177.8	1447.78	-0.435
^{662}KASYTYTVYRDGTK^{675}	37.6	1651.8	-2.282
^{663}ASYTYTVYRDGTK^{675}	133	1523.72	-0.034
^{703}YTAGVSPK^{710}	64.8	821.42	0.091

S9 Table: List of peptides derived from the LCMS analysis of tryptic digested SDS-PAGE gel band (h) from ECR806, D3, VFSN proteins as shown in Fig. 2.

Peptides	MaxQuant Score	Theoretical neutral monoisotopic mass	Mass Error (ppm)
^{56}FTEVQTPK^{63}	138	948.49	0.219
^{66}GIGQVPTYTEGVNLSEK^{80}	123.4	1790.9	0.004
^{99}EMKVEVVSSK^{108}	114.8	1134.59	1.228
^{114}KNVLIAPSK^{121}	105.4	968.6	0.106
^{132}KIPYVYGK^{139}	102.8	966.55	-0.116
^{207}GTFAGFEDTYKR^{218}	133	1390.65	-0.154
^{228}YTPVEEK^{234}	45.7	864.4	0.076
^{228}YTPVEEKQNGR^{238}	71	1319.6	0.025
^{239}MIVIVAK^{245}	90	772.48	0.457
^{239}MIVIVAKK^{246}	99.4	900.58	-0.070
^{246}KYEGDIKDFVDWK^{258}	212	1641.80	-0.079
^{246}KYEGDIKDFVDWKNQR^{261}	99.4	2040.00	-0.312
^{247}YEGDIKDFVDWK^{258}	156	1513.70	0.181
^{253}DFVDWK^{258}	92.7	808.37	-0.094
^{265}TEVKVAEDIASPVTANAIQQFVKQEYEK^{292}	201	3134.6	-0.100
^{269}VAEDIASPVTANAIQQFVK^{287}	311	2000.05	-0.031
^{269}VAEDIASPVTANAIQQFVKQEYEK^{292}	182	2677.35	-0.341
^{288}QEYEKEGNDLTYVLLIGDHK^{307}	56.7	2363.16	1.152
^{288}QEYEKEGNDLTYVLLIGDHKDIPAK^{312}	62.7	2887.46	0.493
^{293}EGNDLTYVLLIGDHK^{307}	236	1685.86	0.088
^{293}EGNDLTYVLLIGDHKDIPAK^{312}	179.8	2210.15	-0.042
^{293}EGNDLTYVLLIGDHKDIPAKITPGIK^{318}	11.2	2819.54	0.415
^{308}DIPAKITPGIK^{318}	84.2	1151.69	0.285
^{319}SDQVYGQIVGNDHYNEVFIGR^{339}	207.5	2409.13	-0.131
^{340}FSCESKEDLK^{349}	90	1184.53	-0.030
^{340}FSCESKEDLKTQIDR^{354}	249.4	1797.85	0.145
^{386}IIKCYDPGVTPK^{397}	69.4	1332.71	0.374
^{412}CYDPGVTPK^{420}	60	978.44	0.217
^{421}NIIDAFNGGISLANYTGHGSETAWGTSHFGTTHVK^{455}	86.4	3659.73	-1.819
^{490}AQKDGKPTGTVAIIASTINQSWASPMR^{516}	115.8	2827.46	-0.405
^{517}GQDEMNEILCEKHPNNIK^{536}	49	2110.97	0.0484
^{517}GQDEMNEILCEKHPNNIKR^{537}	86	2267.07	-0.244
^{535}RTFGGVTMNGMFAMVEK^{551}	146	1874.88	0.203
^{538}TFGGVTMNGMFAMVEK^{551}	207	1718.78	0.001
^{552}YKKDGEK^{558}	58.8	866.44	0.460
^{555}DGEKMLDTWTVFGDPSLLVR^{574}	90.8	2278.13	0.133
^{575}TLVPTK^{580}	52.5	657.4	0.047
^{649}ETVIKTINTNSTK^{661}	151.7	1447.78	0.282

S10 Table: Peptides derived from the LC-MS analysis of trypsin digested purified rRgpAH.

Peptides	MaxQuant Score	Theoretical neutral monoisotopic mass	Mass error (ppm)
^{228}YTPVEEK^{234}	35	864.42	0.078
^{228}YTPVEEKQNGR^{238}	129.7	1319.64	-0.120
^{239}MIVIVAKK^{246}	130.7	900.58	-0.020
^{243}TEVKYEGDIKDFVDWNQR^{260}	144	2457.31	0.057
^{246}KYEGDIKDFVDWK^{258}	267	1641.80	0.113
^{247}YEGDIKDFVDW^{257}	153.8	1513.70	0.292
^{247}YEGDIKDFVDWNQR^{260}	253	1911.91	0.036
^{269}VAEDIASPVTANAIQQFVK^{287}	273.8	2000.05	0.055
^{288}QEYEKEGNDLTYVLLIGDHK^{307}	138	2363.16	0.260
^{288}QEYEKEGNDLTYVLLIGDHKDIPAK^{312}	66.5	2887.46	-0.057
^{293}EGNDLTYVLLIGDHKDIPAK^{312}	276	2210.15	-0.036
^{308}DIPAKITPGIK^{318}	79	1151.69	0.228
^{313}ITPGIKSDQVYGQIVGNDHYNEVFIGR^{339}	86	3018.51	-0.040
^{319}SDQVYGQIVGNDHYNEVFIGR^{339}	219.6	2409.13	-0.079
^{340}FSCESKEDLKTQIDR^{354}	220.7	1797.85	0.113
^{346}EDLKTQIDR^{354}	145.5	1116.57	-0.080
^{350}TQIDRTIHYER^{360}	81.5	1430.72	0.168
^{368}WLGQALCIASAEGGPSADNGESDIQHENVIANLLTQYGYTK^{408}	5	4333.05	2.437
^{409}IIKCYDPGVTPK^{420}	120.6	1332.71	0.148
^{412}CYDPGVTPK^{420}	76	978.44	0.191
^{421}NIIDAFNGGISLANYTGHGSETAWGTSHFGTTHVK^{455}	73	3659.73	-1.581
^{490}AQKDGKPTGTVAIIASTINQSWASPMR^{516}	111.3	2843.45	-0.562
^{487}DGKPTGTVAIIASTINQSWASPMR^{516}	153	2500.27	-0.211
^{517}GQDEMNEILCEK^{528}	217	1407.6	-0.244
^{517}GQDEMNEILCEKHPNNIK^{536}	122.5	2110.97	-0.002
^{537}RTFGGVTMNGMFAMVEK^{551}	162	1874.88	-0.691
^{538}TFGGVTMNGMFAMVEK^{551}	231	1766.76	-0.475
^{538}TFGGVTMNGMFAMVEKYKK^{554}	59	2138.03	0.114
^{555}DGEKMLDTWTVFGDPSLLVR^{574}	39	2294.12	-0.098
^{559}MLDTWTVFGDPSLLVR^{574}	202	1864.93	-0.291
^{581}MQVTAPAQINLTDASVNVSCDYNGAIATISANGK^{614}	27	3436.66	-1.919
^{615}MFGSAVVENGTATINLTGLTNESTLTLTVVGYNKETVIK^{653}	88.3	4085.12	-2.317
^{654}TINTNSTGHHHHHHGK^{669}	127	1813.84	0.109

S11 Table: The Ki of rRgpA-PP, rRgpB-PP, and rKgp-PP against purified gingipains.

**Propeptide**	**rRgpAH** (2.5 nM)	**RgpB** (2.5 nM)	**rKgp** (10 nM)
rRgpA-PP	***K_i*:** 13 nM	***K_i*:** 15 nM	***K_i*:** 13 µM
	**95% CI:** 12 to 14 nM	**95% CI:** 14 to 16 nM	**95% CI:** 12 to 16 µM
rRgpB-PP	***K_i*:** 22 nM	***K_i*:** 29 nM	***K_i*:** 11 µM
	**95% CI:** 20 to 24 nM	**95% CI:** 26 to 32 nM	**95% CI:** 9.0 to 13 µM
rKgp-PP	No inhibition	No inhibition	**K_i:** 38 µM
			**95% CI:** 36 to 39 µM

S12 Table: The K_i of rRgpA-PP and rRgpB-PP against rRgpAH.

rRgpAH Inhibition Kinetics								
**BApNA (mM)**	0.20	0.40	0.60	0.80	1.0	1.2	1.4	1.6
**rRgpA-PP**								
**K_i (nM)**	13	15	15	13	13	14	13	14
**95% CI (nM)**	9-15	13-19	13-18	10-15	12 -14	11-19	11-14	13-15
**rRgpB-PP**								
**K_i (nM)**	26	25	24	24	23	22	23	24
**95% CI (nM)**	24-28	24-26	23-25	26-28	22-24	21-23	22-24	23-25

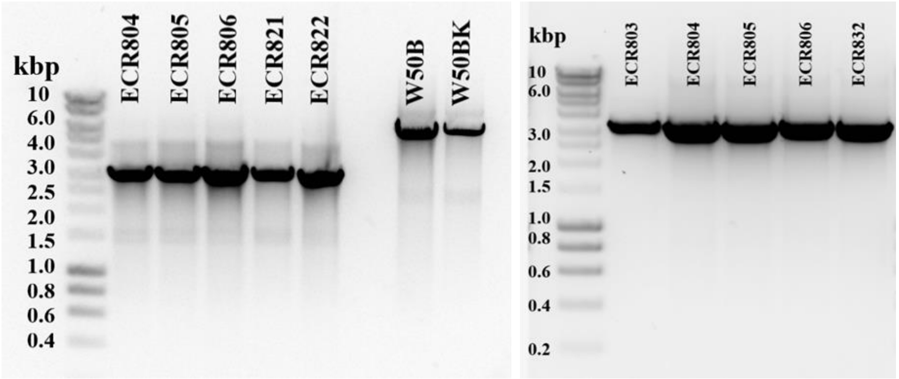

S1 Fig: PCR analysis of transformed *P. gingivalis* genomic DNAs.

pRgpArelease was transformed into *P. gingivalis* to give truncated *rgpA* genes, strain W50 (ECR803), W50B (ECR805), and W50BK (ECR806). ECR803 was transformed with *rgpB::tetQ* (ECR804). *P. gingivalis* W50B and W50BK were transformed with pRgpATH (ECR821 and ECR822 respectively). *P. gingivalis* W50B was transformed with pRgpAH (ECR832). W50B and W50BK are non-recombinant controls. PCR analysis using a reverse primer internal to *ermF* (R01) and flanking forward primer 5’ to the genome recombination point (pgmAR1). Controls used pgmAR1 and RgpAhr3pR.

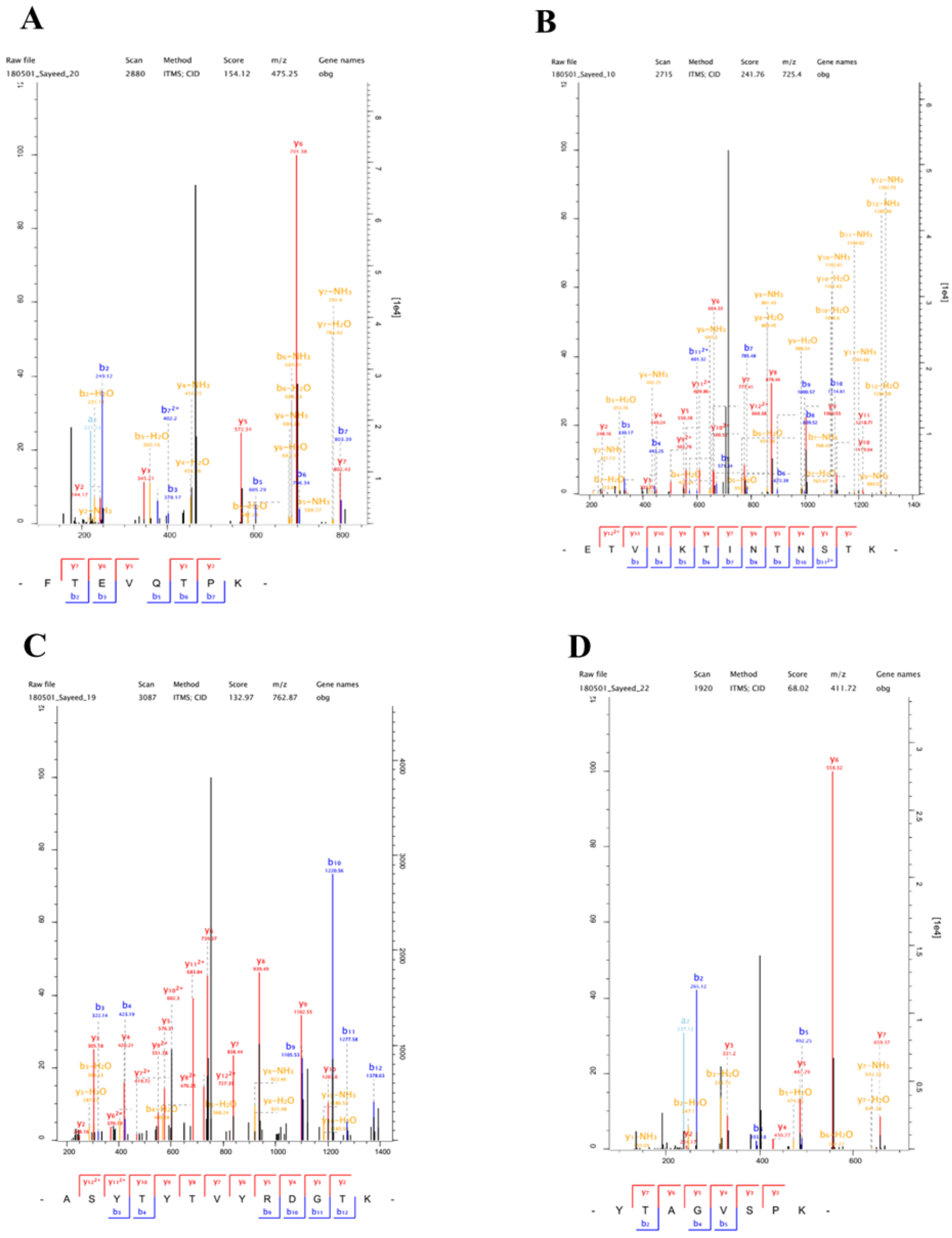

S2 Fig: LC-MS analysis of the rRgpA secreted by ECR806.

(A) The most N-terminal peptide identified corresponds to the predicted rRgpA N-terminus. (B) peptide at the proposed KK cleavage target site of rRgpA. (C) peptide C-terminal to the proposed KK cleavage target site of rRgpA. (D) peptide near the ABM2 C-terminus. Panel (D) indicates that rRgpA did not release due to cleavage at the KK site, rather cleavage of rRgpA occurs within KVCVDYIPDGVA at the polypeptide C-terminus.

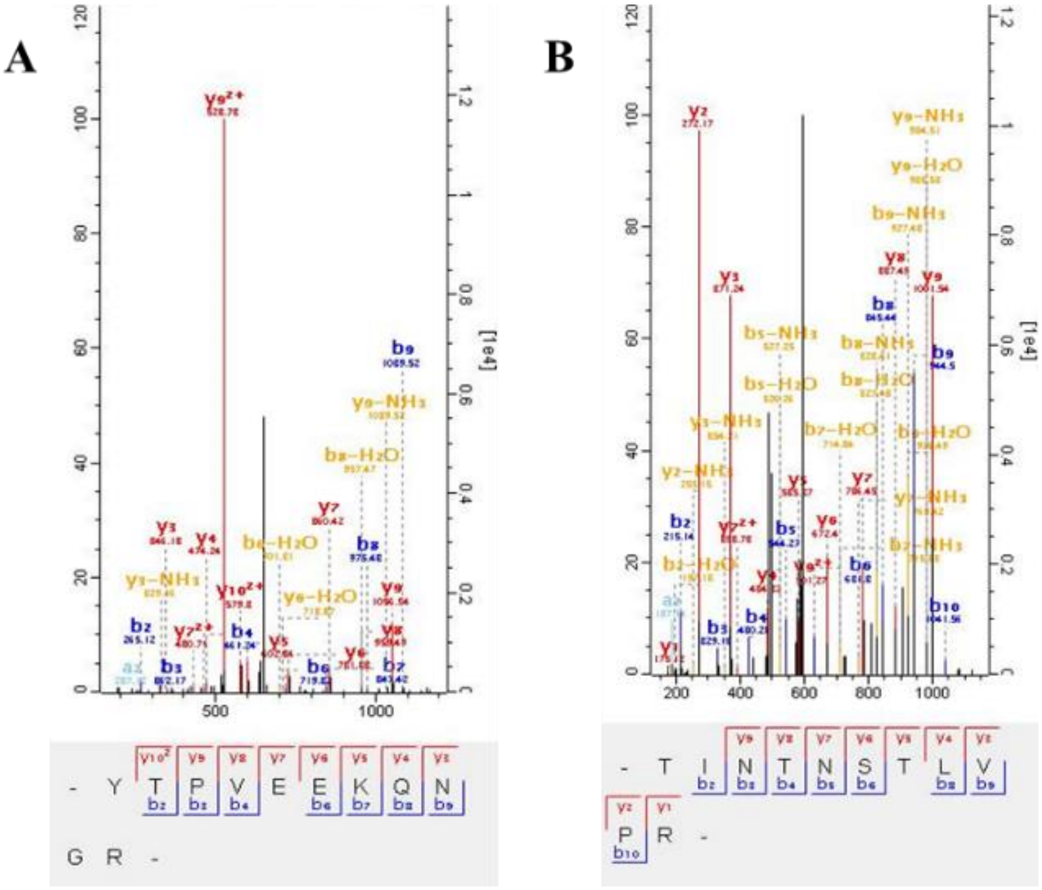

S3 Fig: The LC-MS analysis of trypsin digested peptides of rRgpATH in the ECR821 VFSN band j (Fig 2.). The mature rRgpATH N-terminus fragment was identified (A). The most C-terminal peptide identified was cleaved within the thrombin recognition site (B). No peptide with a His affinity tag was identified.

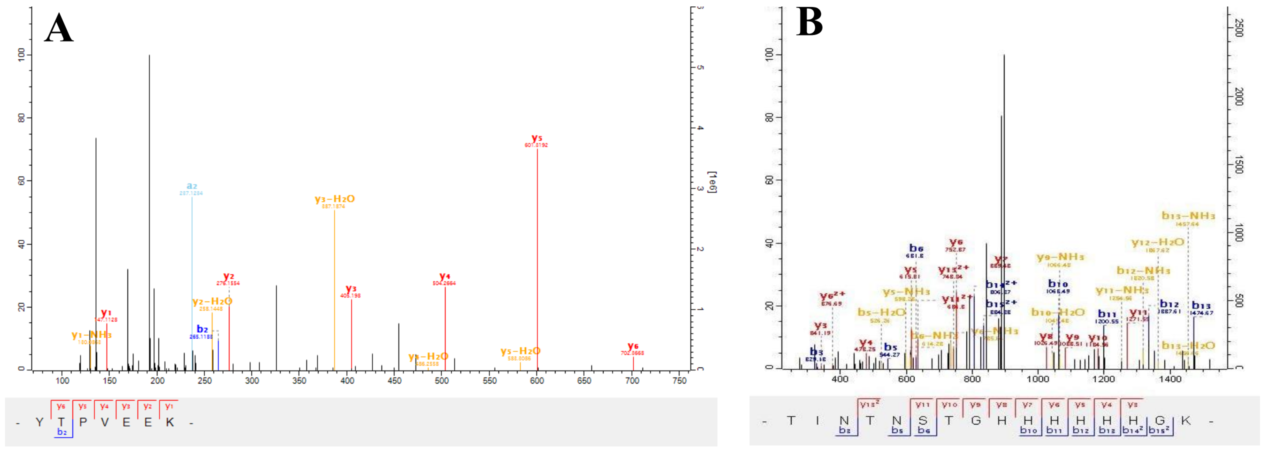

S4 Fig: The LC-MS analysis of tryptic digested rRgpAH peptides.

N-terminal fragment (A) and the C-terminal fragment (B) confirming the presence of the His affinity tag and a Lys residue at the C-terminus.

A

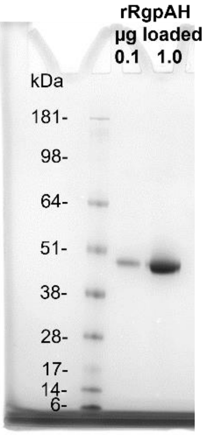

B

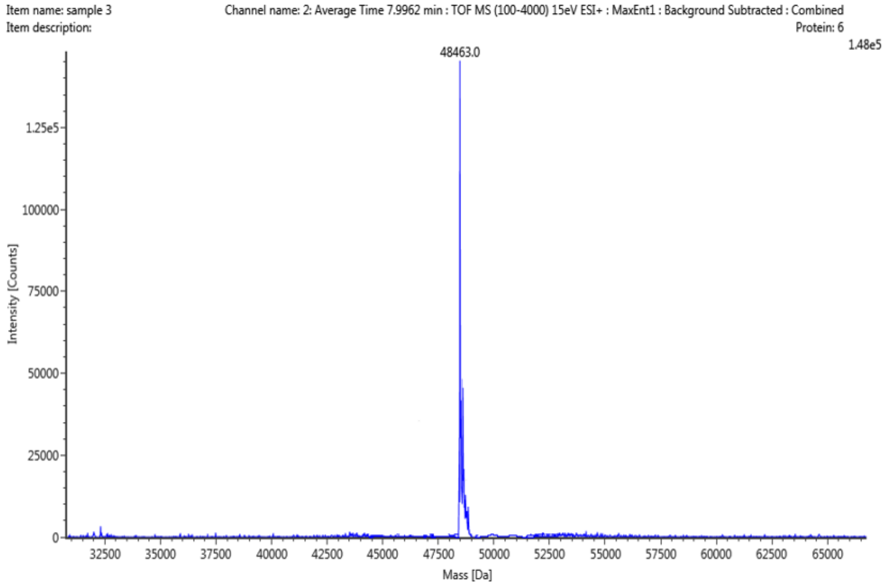

S5 Fig: SDS-PAGE and MS analysis of purified rRgpAH.

(A) Denaturing SDS-PAGE of purified rRgpAH. Lanes were loaded with 0.1 µg or 1.0 µg of rRgpA as indicated. The gel was a 4-12% Nu-PAGE gel run using MOPS running buffer. See Blue Plus molecular mass standards (Invitrogen) are indicated to the left (B) The deconvoluted mass spectrum of purified rRgpAH.

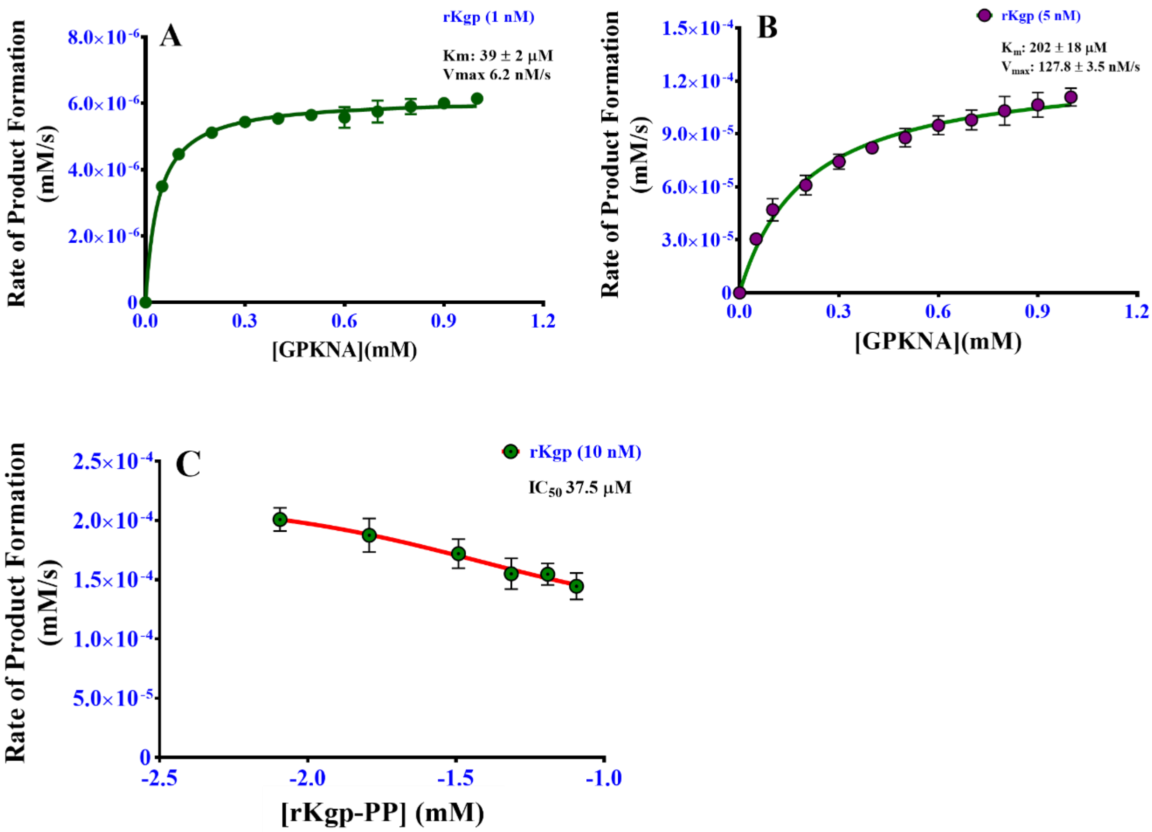

S6 Fig: The kinetics of rKgp using substrate GPKNA.

(A) The kinetics of rKgp with no glycylglycine as an assay reagent. (B) The kinetics of rKgp using 180 mM of glycylglycine as an assay additive. (C) Dose-dependent effect of rKgp-PP against purified Lys-gingipains rKgp. Reactions for (B) and (C) were carried out in a 200 µL mixture of Tris 50 mM, NaCl 150 mM, $CaCl_2$ 5 mM, pH 8, 20 mM Cys, 180 mM glycylglycine, rKgp (5 nM for B, and 10 nM for C), varying concentrations of rKgp-PP, 1.0 mM GPKNA. Assay (A) was carried out by replacing glycylglycine with Milli Q water.

Original gel and blot images

Figure 2: Panel (a)

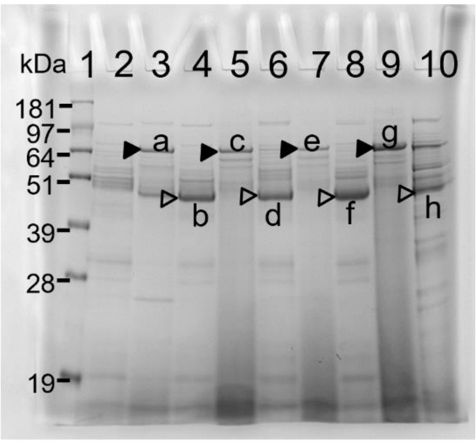

Figure 3: Panel (b)

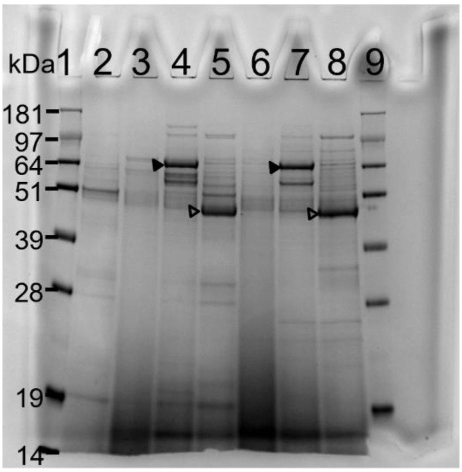

Figure 3: Panel (c)

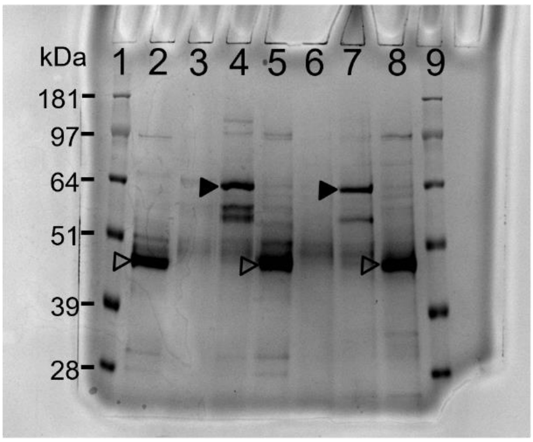

Figure 3: Panel d(i)

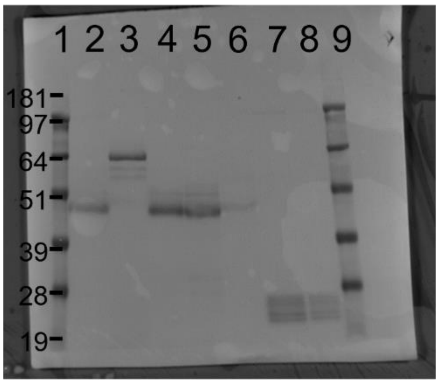

Figure 3: Panel d(ii)

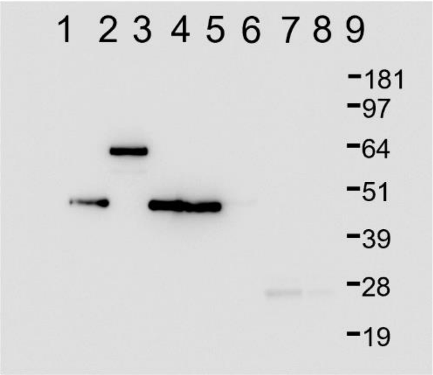

S7 Fig: Original gel and blot images
